# Factors associated with the uptake and utilisation of diabetic retinopathy screening services in sub-Saharan Africa: A scoping review

**DOI:** 10.1371/journal.pone.0315367

**Published:** 2024-12-13

**Authors:** Iheanyi Oby Nwaoha, Albain Ayime Balibuno, Nuha Ibrahim

**Affiliations:** Faculty of Education and Health Sciences, Department of Public Health, School of Medicine, University of Limerick, Limerick, Ireland; College of Medicine, University of Nigeria, NIGERIA

## Abstract

**Introduction:**

Diabetic Retinopathy (DR) is a microvascular complication of chronic Diabetes that can lead to visual impairment if left untreated. While concerted efforts have been made to develop screening modalities to facilitate the early detection of Diabetic Retinopathy in sub-Saharan Africa, little is known about the factors impacting the optimal use of these screening services. This paper aims to identify and highlight factors associated with the access of Diabetic Retinopathy screening services from patient and service provider perspectives.

**Methodology:**

This scoping review was conducted using the Arksey and O’Malley (2005) framework. A comprehensive search of peer-reviewed articles and grey literature was conducted from May 2023 to June 2023. Electronic databases searched include Medline, Embase, PubMed, CINAHL Complete, APA PsycINFO, Web of Science, and African Journal Online (AJOL). Two reviewers independently screened the retrieved records for eligibility, and relevant data was extracted from the included studies. A descriptive overview of key findings was provided, and the 5As conceptual framework of access to healthcare was used to map the identified factors.

**Results:**

The search strategy yielded 873 records. Of those, 19 studies met the criteria for inclusion. Health literacy and duration of Diabetes were reported in 12 and 9 studies as the most common factors associated with DR screening services access. Similarly, age at onset and inadequate referral by healthcare providers were cited as significant determinants of DR screening access in 7 studies, respectively.

**Conclusion:**

The 5As framework of access to healthcare aids our understanding of factors associated with the access of DR screening from patient and service provider standpoints. To address these issues, there is a need for more research on this topic to design effective DR screening services in the region.

## 1 Introduction

Diabetes Mellitus (DM) remains a significant public health challenge globally. Broadly classified into Type 1, Type 2, and gestational Diabetes, the International Diabetes Federation (IDF) estimates that about 537 million people (approximately 10.5% of the adult population worldwide) between the ages of 20 to 79 have Diabetes and this figure is forecast to markedly increase by 46% to 783 million by 2045 [1]. The burden of Diabetes Mellitus is unevenly distributed globally, with inordinately higher prevalence rates recorded in low- and middle-income countries [2]. Even in high-income countries, there are stark socio-economic disparities in the distribution of non-communicable diseases like Diabetes across households of different income brackets [3]. For sub-Saharan Africa, which is currently grappling with a myriad of issues ranging from poverty, malnutrition and infectious diseases, the Diabetes epidemic poses enormous challenges to the health and socio-economic well-being of its teeming population [4]. In Africa, the age-standardized prevalence of Diabetes is estimated to be approximately 7.2% among adults aged 20–79 years [1]. Moreover, the probability of developing diabetic microvascular complications is relatively higher in people of African descent due to a high incidence of comorbidities such as hypertension, poor metabolic control, and a probable genetic predisposition to the disease condition [5]. Economically, sub-Saharan Africa is also known to contribute the least to the total healthcare expenditure for annual Diabetes care worldwide [6].

Diabetic Retinopathy (DR), a sight-threatening complication of long-standing Diabetes is a leading cause of preventable blindness [7]. In its initial stages, DR may be completely asymptomatic but can progressively result in sudden, irreversible loss of vision if left unchecked. As the progression of DR is inherently linked to the duration of DM, routine DR screening is considered essential to reduce DR progression [8]. There is often a delay between the onset of Type 2 diabetes and its diagnosis. As a result, people with Type 2 diabetes may already have diabetic retinopathy (DR) at the time of diagnosis. This delay is less common in persons with Type 1 diabetes [9]. As part of the guidelines for diabetic eye care of 2017, the International Council of Ophthalmology (ICO) and the American Diabetes Association (ADA) recommend routine eye examinations for persons with Type 1 Diabetes, 5 years post-diagnosis, while people with Type 2 Diabetes should be screened at the time of diagnosis [10]. Depending on the degree of DR at baseline, the American Academy of Ophthalmology also advocates that follow-up exams be conducted yearly or earlier [11].

In addition to ensuring timely intervention, the judicious utilisation of DR screening services is known to be cost-effective [12]. Beyond detecting vision-threatening DR, regular retinal screening can provide some insight into a diabetic patient’s current state of health, as DR can serve as an essential biomarker in identifying subsets of the population at risk of developing other diabetic complications such as nephropathy and neuropathy [13]. The absence of a cohesive infrastructure for ophthalmic services in resource-poor settings has been identified as a major impediment to routine Diabetic Retinopathy screening [14]. For instance, Africa is estimated to have an ophthalmologist-to-population ratio of roughly 2.5 to 1 million, which means that patients with Diabetes cannot quickly receive the retinal follow-up they need [15].

To set up an effective DR screening service (DRSS), it is crucial to have a grading system in place to recognise points of intervention as the disease progresses. One of the earliest attempts to provide a standardised classification scheme for DR was the Airlie House classification 1968 [12]. The Early Treatment of Diabetic Retinopathy Study (ETDRS) developed a refined version of the former classification, and introduced the term clinically significant macular edema (CSME) to depict the loss of vision associated with DR and this soon became the gold standard [16]. Subsequently, in 2003, the international classification of Diabetic Retinopathy (ICDR) introduced the International Clinical Disease Severity Scale for Diabetic Retinopathy providing a detailed 5-stage classification of the condition as follows: no apparent retinopathy; mild non-proliferative Diabetic Retinopathy (NPDR); moderate NPDR; severe NPDR; and Proliferative Diabetic Retinopathy (PDR) and 3 categories of Diabetic macular edema: mild DME, moderate DME, Severe DME. DR is designated as vision-threatening (VTDR) during the severe NPDR, and PDR stages [17].

Like other screening services in public health, setting up an efficient DR screening programme involves determining the population eligible for screening, developing a system of invitation and information sharing to eligible cohorts and using the most suitable tests for the screening exercise. It also includes a referral pathway for all screen positives and a feedback loop to report all screen negatives to individuals. In some cases, it goes as far as ensuring accurate diagnosis of true cases, providing the right intervention and follow-up as required [18]. According to the St Vincent Declaration of 1989, a systematic screening programme should aim to achieve a sensitivity, specificity, and coverage of ≥ 80% respectively [19]. In 1968, Wilson and Junger published ten criteria for a public health screening program, most of which are still valid today [20].

Establishing a systematic DR screening programme in underserved communities is quite daunting [21]. The absence of essential components of the health system makes it increasingly difficult for proper service coordination to meet the needs of people living with Diabetes in these settings. Impediments to service provision include inequitable distribution of healthcare providers between urban and rural areas, lack of incentive to encourage service providers to move into underserved communities, inadequate staff training, absence of basic amenities like good roads, internet access, and housing [22]. With growing demand and limited supply of services, DR screening is now left in the hands of non-governmental organisations and private institutions. Where present, these services are often fragmented and siloed precluding integration within the wider network of services needed for optimal diabetic care and management [23]. They may also come at a high cost, creating undue financial hardship to target users. To reach out to people living with Diabetes in these communities, a situational analysis of all components of the health system from the perspective of patients and service providers is necessitated.

The 5As framework of access to healthcare by Levesque et al (2013) provides invaluable insights into the concept of healthcare accessibility in the face of limited resources [24,25]. While previous literature reviews conducted on this topic in sub-Saharan Africa have assessed Diabetic Retinopathy screening services (DRSS) from the health systems/supply and demand point of views [22], the rationale behind this review is to achieve a wider view of the contextual factors associated with DRSS access in the region. The 5As framework stands out for this purpose because it factors in both healthcare providers and patient/population perspectives on accessibility [26]. It describes access as the ability to identify, seek, reach, obtain, or utilize healthcare services, ensuring that the needs for these services are met. The framework defines five key dimensions of accessibility as follows: Approachability, Acceptability, Availability and accommodation, Affordability, and Appropriateness. These dimensions interact with five corresponding abilities within populations to create access. The related abilities are: Ability to perceive, Ability to seek, Ability to reach, Ability to pay, and Ability to engage [25].

Based on the foregoing, conducting a scoping review of published literature on Diabetic Retinopathy screening services access in sub-Saharan Africa is pertinent.

### 1.1 Study aim

This study aims to review the contextual issues associated with the access of screening services for Diabetic Retinopathy in sub-Saharan Africa.

#### 1.1.1 Review objectives

- To comprehensively map the extent of the existing literature on access to Diabetic Retinopathy screening services in sub-Saharan Africa.
- To highlight the factors associated with the access of Diabetic Retinopathy screening services from service user and service provider standpoints.

## 2 Methodology

This review was conducted in line with the methodological framework by Arksey and O’Malley [27] and reported using the Preferred Reporting Items for Systematic Reviews and Meta-Analyses (PRISMA ScR) extension guidelines for scoping reviews. (See S2 Appendix). A review protocol was developed a priori and registered on the Open Science Framework: https://doi.org/10.17605/OSF.IO/HDRZJ

### 2.1 Framework

The Population, Concept and Context (PCC) framework for conducting scoping reviews recommended by Joanna Briggs Institute (JBI) in 2013 was used to develop the review question [28]. Based on this method of evidence synthesis, we deduced the following and posed the review question below:

**Population of interest:** People living with Diabetes

**Concept**: Access of Diabetic Retinopathy screening services (DRSS).

**Context:** sub-Saharan Africa

#### 2.1.1 Review question

What are the factors associated with the access of Diabetic Retinopathy screening services for people living with Diabetes in sub-Saharan Africa?

### 2.2 Search strategy

To answer the review question, the Elton B. Stephens Company (EBSCO) host platform was accessed through the University of Limerick Glucksman Library website from 29^th^ May 2023 to 30^th^ June 2023. Electronic databases searched include MEDLINE with full text, PubMed, Embase, APA PsychINFO, Web of Science, CINHAL Complete, and Africa Journals Online. These databases were searched to ensure comprehensive coverage, capture discipline-specific studies, and avoid selection bias. There was no publication time limit on the searches because of the paucity of available studies on the topic after a preliminary search.

Searches were run for these terms in the title, abstract and full-text fields. To guide our search properly, we searched related terms for our key concept “Diabetic Retinopathy Screening Services” under the medical subject headings MeSH terms for MEDLINE, CINAHL subheadings for CINAHL complete using a combination of Boolean operators (AND, OR, NOT) and other search terms to narrow or broaden the search as required. Keywords in the studies we found were reviewed and references lists were scanned to expand, refine, and modify our search terms as required. Utmost care was taken to document our search strings after each session to keep track of all queries. Retrieved articles were exported to the reference manager, Endnote, for proper filing. Hand searches of grey literature were equally conducted.

### 2.3 Study selection

Articles found on the electronic research databases were imported to the artificial intelligence software application, Rayyan, where they were scrutinized for duplicates [29]. These duplicates were subsequently removed. Two authors (IN and AB) independently screened the title and abstracts of the remaining studies identified from the databases on Rayyan. Articles were assessed using a set of eligibility criteria (outlined below), after which conflicts were discussed. These conflicts were resolved by consensus and through arbitration of a review tiebreaker (NI) as required. The Preferred Reporting Items for Systematic review and Meta-Analysis (PRISMA) flow diagram was then created to illustrate the entire process of study selection. Reasons for exclusion of articles at the full-text stage were provided.

#### 2.3.1 Inclusion criteria

- Population-based or hospital based primary studies (quantitative, qualitative or mixed methods) and any study design, conducted in sub-Saharan Africa and published in English.
- Studies reporting factors associated with the access of DR screening from patient and/or service provider perspectives.

#### 2.3.2 Exclusion criteria

- Studies that do not involve human subjects, including editorials, case reports, opinion papers, conference abstracts, review protocols and systematic reviews.
- Studies evaluating barriers to eye care in general, without focusing on Diabetic Retinopathy screening, and studies assessing screening obstacles for Diabetes Mellitus without mentioning Diabetic Retinopathy screening hurdles specifically.
- Studies focusing solely on prevalence, diagnosis, medical, or surgical/para-surgical management of Diabetic Retinopathy.
- Articles not published in English
- Studies not conducted in sub–Saharan Africa.

### 2.4 Data extraction

The full text of the included studies was read, and relevant data was extracted using Microsoft Excel®. Each article was assigned a number for easy identification. Information extracted include author, title, year of publication, study location (country and setting), study population and sample size, research methods used, emerging themes and 5As dimensions. Two authors (IN and NI) worked independently on data extraction, and iteratively identified emerging themes. Thematic synthesis was conducted in three overlapping steps involving line by line coding of the findings from the included studies, grouping these codes into descriptive themes, and generating analytical themes from these categories using the 5As framework [30]. The data extraction table was pilot tested to refine the categories, ensure clarity and promote consistency across the reviewers for more reliable data. A risk of bias or critical appraisal of the included studies was not conducted in this scoping review as it is considered optional [31].

### 2.5 Collating, summarizing and reporting the results

A narrative account of key findings from the included literature was summarized after collating and analysing the extracted data for emerging themes. To shed more light on some contextual factors related to the access of DR screening services in sub-Saharan Africa, we used the 5A’s conceptual framework of access to healthcare by Levesque et al (2013) to map and explain the emerging themes from the studies.

## 3 Results

Overall, the searches generated 873 articles, out of which 405 duplicates were removed. 468 titles and abstracts were screened by the reviewers using the designated eligibility criteria. Based on the information provided in the titles and abstracts, 445 (95%) articles did not meet the inclusion criteria and were excluded. Reasons for exclusion include:

Wrong outcome (n = 397), Wrong population (n = 41), non-human subject (n = 3), Review Protocol (n = 3), Not English (n = 1). (The abstract for this study was available in English but it was excluded later because it did not meet the inclusion criteria).

**Fig 1:**
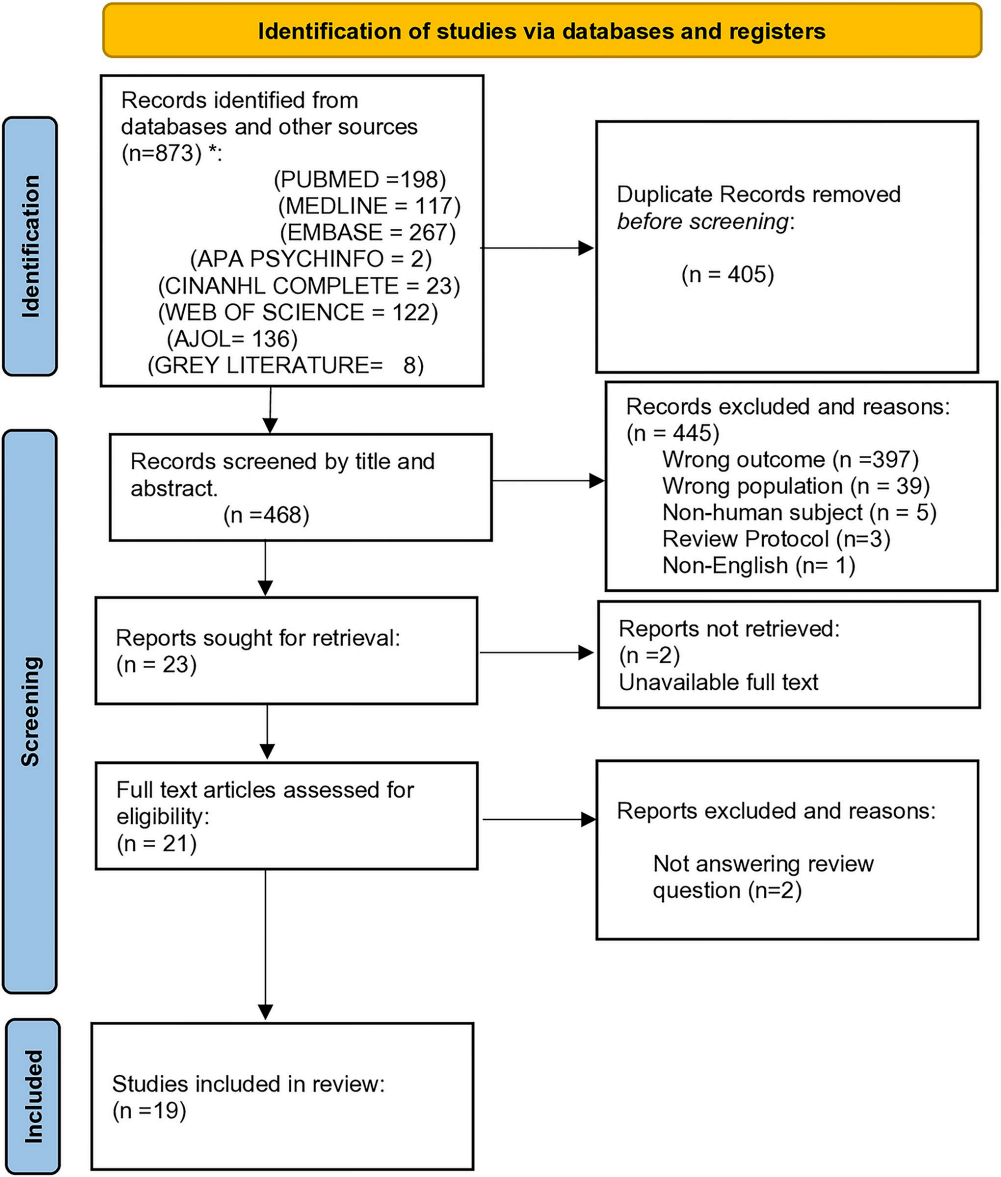
PRISMA flow diagram of study selection.

Afterwards, we sought to retrieve the remaining 23 articles for full text screening. The full text of 2 articles were unretrievable, so the other 21 articles were assessed for eligibility. After reading the full text, 2 extra articles were excluded because they did not answer the review question. See Fig 1.

### 3.1 General characteristics of included studies

**Fig 2:**
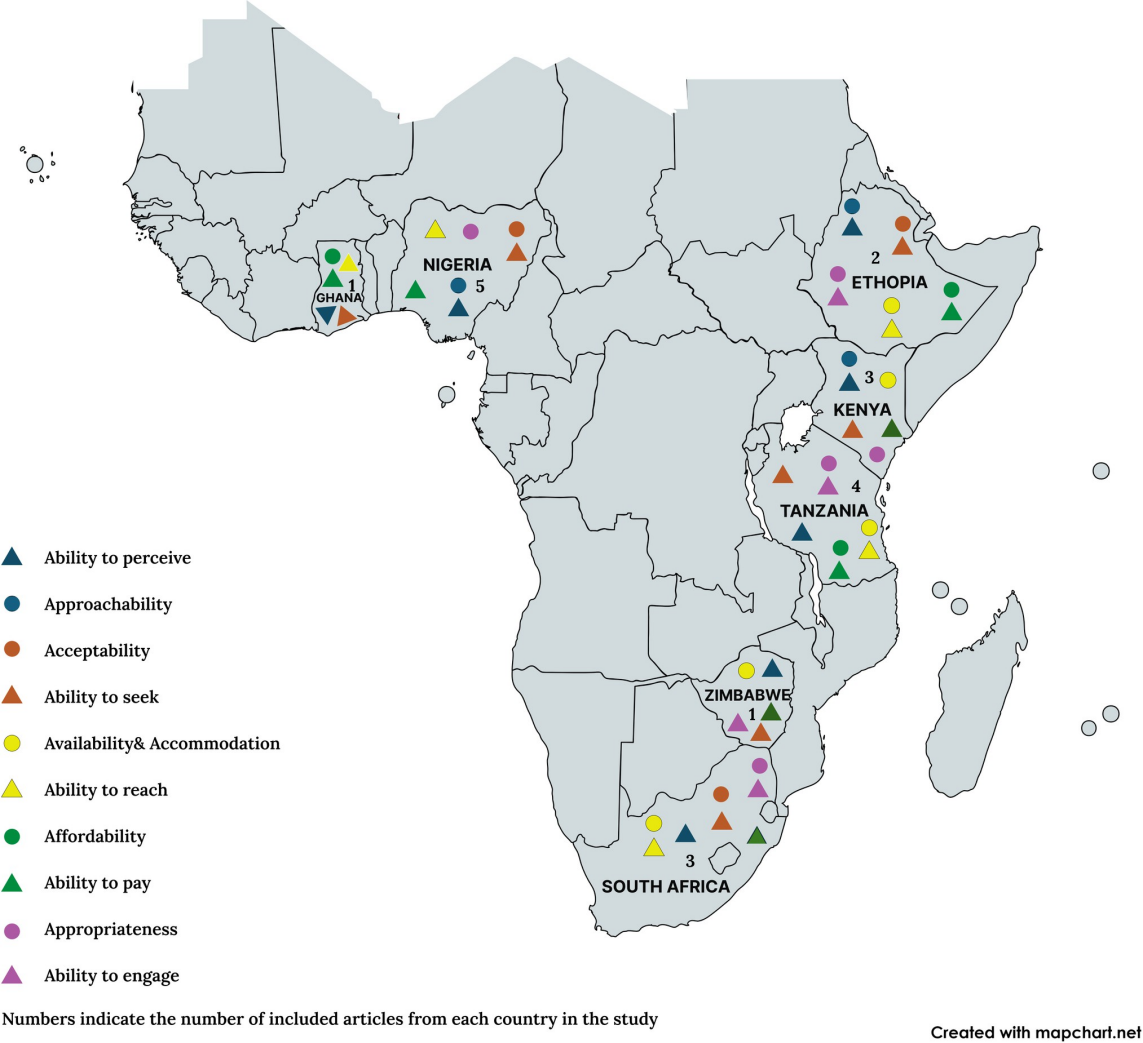
Geographical distribution of included studies and 5A dimensions as reported.

Nineteen studies published in different scientific journals between 2007 and 2022 were included in this review. All the studies were observational studies using cross-sectional study design to assess the factors associated with the access of DRSS. Three of the studies [#6, #11, #19] investigated DRSS access from the service provider’s point of view, while 15 articles [#1, #2, #3, #4, #5, #7, #8, #9, #10, #12, #13, #14, #15, #16, #18] assessed from the patients’ standpoint. Study #17 explored both patient and service provider perspectives. Of all 19 studies, 14 studies were quantitative studies relying on interviewer administered questionnaires, 2 studies [#11 and #17] were qualitative studies involving face-face-face semi-structured interviews while 3 articles [#5, #9 and #10] used mixed methods. 10 of the studies used systematic random sampling technique to recruit study participants, while 6 of the studies resorted to convenience sampling. Two studies [#17 and #19] utilized purposive sampling while study #6 relied on proportionate allocation. The sample sizes ranged from 27 to 424 participants. With regards to country and setting, the studies were conducted in various primary, district and tertiary healthcare facilities across sub-Saharan Africa (5 in Nigeria, 1 in Ghana, 2 in Ethiopia, 4 in Tanzania, 3 in Kenya, 3 in South Africa, 1 in Zimbabwe). See Table 1 and Fig 2.

**Table 1:** Included studies, emerging themes and 5A dimensions/abilities.

NO (#)	TITLE	AUTHORS AND YEAR	COUNTRY	SETTING	STUDY DESIGN	STUDY POPULATION	THEMES	5A DIMENSIONS AND ABILITIES
1	Compliance with Diabetic Retinopathy screening in a Nigerian tertiary hospital	Onakpoya *et al*. 2015 [32]	Nigeria	Endocrinology Outpatient Clinic and the Ophthalmology Outpatient Unit of the Wesley Guild Hospital, Ilesha.	QUANTITATIVE: cross-sectional study using Questionnaires	179 Type 2 Diabetes patients who attended the Endocrinology Outpatient Clinic.	Health-related factors, service coordination	• Ability to perceive, • Appropriateness
2	Diabetic Retinopathy and retinal screening awareness amongst female diabetic patients at a day hospital diabetic clinic in Cape Town, South Africa	Mkhombe and Clarke-Farr 2021 [33]	South Africa	Khayelitsha Site B, day clinic located in Cape town	QUANTITATIVE: Descriptive observational study using structured survey questionnaires	149 female patients between 30 and 60 years of age, who attended the diabetic clinic at the day hospital.	Health literacy, demographic factors, service accessibility, service coordination	• Ability to perceive, • Ability to seek, • Availability & Accommodation • Appropriateness
3	Improving Diabetic Retinopathy screening in Africa: Patient satisfaction with teleophthalmology versus ophthalmologist-based screening	Kurji *et al*. 2013 [34]	Kenya	Nairobi (Patients were selected from a database at a one-stop multidisciplinary Diabetes clinic)	QUANTITATIVE: Cross-sectional observational study using Structured patient satisfaction questionnaire with a five-point Likert scale	57 patients who had received both an ophthalmologist-based and teleophthalmology screening examination.	Service accessibility, Service coordination	• Availability & Accommodation • Appropriateness
4	Prevalence, Awareness and Determinants of Diabetic Retinopathy in a Screening Centre in Nigeria	Kizor-Akaraiwe *et al*. 2016 [35]	Nigeria	Enugu (undisclosed Diabetic Retinopathy Screening Centre)	QUANTITATIVE: cross-sectional study using interviewer-administered questionnaires	95 consenting diabetic patients who visited a DR screening Centre in Enugu.	Demographic factors, health literacy, heath related factors, financial factors, service reach, service coordination	• Ability to perceive, • Ability to seek, • Ability to pay, • Approachability • Appropriateness
5	Gender bias within a Diabetic Retinopathy screening programme in Tanzania	Clel *et al*. 2022 [36]	Tanzania	Diabetic clinics under Kilimanjaro Diabetic Programme (KDP) in the Kilimanjaro region of northern Tanzania.	MIXED METHODS: cross-sectional study using Questionnaire and face-to-face interviews	340 patients registered with the Kilimanjaro Diabetic Programme (KDP)	Health literacy, demographic factors, financial factors, health beliefs, residence, mobility & social factors, service costs,	• Ability to perceive, • Ability to seek, • Ability to reach, • Ability to pay, • Affordability
6	Knowledge, attitude, and practice of Diabetic Retinopathy among physicians in Northwestern Nigeria	Abdulsalam *et al*. 2018 [37]	Nigeria	A multi-center study involving 4 tertiary hospitals	QUANTITATIVE cross-sectional study using questionnaires	110 general practitioners (GPs), residents, and consultants	Health literacy, screening competency, service coordination	• Ability to perceive, • Acceptability, • Appropriateness
7	Utilisation of eye health services and Diabetic Retinopathy: a cross-sectional study among persons living with Diabetes visiting a tertiary eye care facility in Ghana	Akrofi *et al*. 2021 [38]	Ghana	Korle Bu Teaching Hospital (KBTH)	QUANTITATIVE Cross-sectional study using questionnaires.	360 Patients with Diabetes visiting the endocrine clinic at KBTH	Demographic factors, health related factors, financial factors, residence, mobility & social factors, service costs	• Ability to perceive, • Ability to seek, • Ability to pay, • Ability to reach, • Affordability
8	Knowledge about Diabetic Retinopathy, eye check-up practice and associated factors among adult patients with Diabetes mellitus attending at debark hospital, Northwest Ethiopia	Assem *et al*. 2020 [39]	Ethiopia	Debark Hospital	QUANTITATIVE: Cross-sectional study using a structured face to face interviewer administered questionnaire.	238 diabetics attending Debark hospital.	Demographic factors, health related factors, financial factors health literacy, residence, mobility & social factors, service reach	• Ability to perceive, • Ability to seek, • Ability to pay, • Ability to reach, • Approachability
9	Factors associated with Diabetic Retinopathy screening and regular eye checkup practice among diabetic patients attending Felege Hiwot Specialized Hospital	Fekadu *et al*. 2022 [40]	Ethiopia	Felege Hiwot Specialized Hospital	MIXED METHOD Cross-sectional study using semi-structured questionnaire and interview	424 diabetics attending Felege Hiwot diabetic clinic	Demographic factors, psychological factors, health related factors, health literacy, financial factors, residence mobility & social factors, service accessibility, service costs, service coordination	• Ability to perceive, • Ability to seek, • Ability to reach, • Ability to engage, • Availability & Accommodation, • Affordability • Appropriateness
10	A needs assessment of people living with Diabetes and Diabetic Retinopathy	Hall *et al*. 2016 [41]	Tanzania	5 rural district hospitals in the Kilimanjaro region (MULTICENTRE STUDY)	MIXED METHODS Cross-sectional involving Standardized interview questionnaire and 5 quantitative Psychometric measures)	27 people living with Diabetes invited to attend DR screening at the district hospitals.	Financial factors, health related factors, health literacy, residence, mobility, & social factors, psychological factors, service accessibility, service costs	• Ability to perceive, • Ability to reach, • Ability to pay, • Ability to engage, • Accommodation and availability • Affordability
11	Diabetes and Diabetic Retinopathy Management in East Africa: Knowledge, Attitudes, and Practices of Hospital Staff in Kenya	Kupitz *et al*. 2014 [42]	Kenya	Kenyatta National hospital (KNH) (HOSPITAL-BASED-TERTIARY)	QUALITATIVE Cross-sectional study using semi-structured interviews	46 participants working in the Departments of Internal Medicine or Ophthalmology at KNH.	Service reach, screening competency, service co-ordination, health related factors	• Ability to perceive, • Approachability • Acceptability • Appropriateness
12	Reasons for poor follow-up of Diabetic Retinopathy patients after screening in Tanzania: a cross-sectional study	Mtuya *et al*. 2016 [43]	Tanzania	Kilimanjaro Diabetic Programme (18 diabetic clinics across 7 districts in Kilimanjaro Region) (MULTI-CENTRE STUDY)	QUANTITATIVE cross-sectional study using structured questionnaires	294 patients referred to KCMC eye department after a screening event.	Financial factors, psychological factors, health literacy, health beliefs, residence, mobility & social factors, service accessibility, service costs, service coordination	• Ability to perceive, • Ability to reach, • Ability to engage, • Availability & accommodation • Affordability • Appropriateness
13	Compliance with eye screening examinations among diabetic patients at a Tanzanian referral hospital	Mumba *et al*. 2007 [44]	Tanzania	Kilimanjaro Christian Medical Centre Hospital (KCMC)	QUANTITATIVE cross-sectional study closed ended questionnaire	316 adult patients attending the diabetic clinic at KCMC.	Demographic factors, health related factors, health literacy, financial factors	• Ability to perceive, • Ability to seek, • Ability to pay
14	Predictors of uptake of eye examination in people living with Diabetes mellitus in three counties of Kenya	Mwangi *et al*. 2017 [45]	Kenya	Kirinyaga, Nakuru, Nairobi (3 Diabetes outpatient clinics were selected in each county) MULTI-CENTRE STUDY	QUANTITATIVE cross-sectional study using interview structured questionnaires	270 eligible adult patients known to have Diabetes, resident in the county, receiving services at participating outpatient Diabetes clinics.	Demographic factors, health related factors, health literacy, service coordination	• Ability to perceive, • Ability to seek, • Appropriateness
15	Awareness and attitude of diabetic patients on diabetic eye complications in Port-Harcourt, Nigeria	Nathaniel and Adio 2015 [46]	Nigeria	University of Port Harcourt Teaching hospital (UPTH)	QUANTITATIVE: cross sectional using semi-structured interview questionnaires	225 subjects drawn from those attending the endocrinology clinic	Health literacy and health related factors, service reach,	• Ability to perceive, • Approachability
16	Determinants of previous dilated eye examination among type II diabetics in Southwestern Nigeria	Onakpoya *et al*. 2010 [47]	Nigeria	Obafemi Awolowo University Teaching Hospital	QUANTITATIVE Cross-sectional study using a structured protocol administered to attendees.	83 diabetics attending the diabetic clinic.	Demographic factor, health related factors, health literacy, residence, mobility & social factors, service coordination	• Ability to perceive • Ability to seek, • Ability to reach, • Appropriateness
17	Exploring Factors Associated with Diabetic Retinopathy Treatment Compliance Behaviour in Cape Town, South Africa	Wentzel and McHiza 2021 [48]	South Africa	Primary health care facilities in the Northern/Tygerberg sub-Structure (NTSS) public health care system of Cape Town	QUALITATIVE cross-sectional study using semi-structured interviews	13 patients living with Diabetes who were attending day hospitals in the NTSS primary care facility, and 2 key informants consisting of service providers.	Health related factors, health literacy, financial factors, health beliefs, psychological factors, residence, mobility & social factors, service accessibility, service coordination	• Ability to perceive, • Ability to reach, • Ability to pay, • Ability to engage, • Availability & Accommodation • Appropriateness
18	Uptake of Screening for Diabetic Retinopathy and Associated Factors among Adults with Diabetes Mellitus Aged 18–65 Years: A Descriptive Cross-Sectional Study	Mukona *et al*. 2020 [49]	Zimbabwe	Parirenyatwa Group of Hospitals Out Patients’ Department (OPD)	QUANTITATIVE cross-sectional study using interviewer administered questionnaires.	83 adults aged 18–65 years with Diabetes at Parirenyatwa Group of Hospitals.	Financial factors, demographic factors, health literacy, psychological factors, health beliefs, service accessibility	• Ability to perceive, • Ability to seek, • Ability to pay, • Ability to engage, • Availability and Accommodation
19	Competency level assessment of healthcare practitioners in managing Diabetes and diabetic eye disease in the district health system of Limpopo province, South Africa	Abdool *et al*. 2020 [8]	South Africa	Voortrekker district hospital, public health institutions of Waterberg district and Mankweng Hospital complex (Capricorn district) in Limpopo province	QUANTITATIVE Cross sectional study using Questionnaires	74 primary healthcare nurses (PHC nurses), optometrists, ophthalmic nurses, and medical officers (MOs)	Screening competency, Service coordination	• Acceptability • Appropriateness

### 3.2 Thematic synthesis

Seven themes (health literacy, health-related factor, health beliefs, demographic factors, psychological factors, financial factors; residence, mobility, and social factors) emerged from the review in relation to the patients while five themes (service reach, service cost, screening competency, service accessibility and service coordination) emerged in relation to service providers.

Patient-related themes were categorized using the five abilities of the population in the 5As conceptual framework as follows:

### Ability to perceive

- Health Literacy: This was one of the popular themes emerging from 63% of the studies [#2, #4, #5, #8, #9, #10, #13, #14, #15, #16, #17, #18]. These studies assessed the level of awareness and knowledge of Diabetes, Diabetic Retinopathy, and importance of periodic retinal screening among patients living with Diabetes. Majority of the studies showed moderate to high levels of awareness and knowledge of Diabetic Retinopathy, while in 4 studies [#8, #10, #16, #17], this was reportedly low.

- Health-related Factors: According to the studies, duration of Diabetes was one of the single largest determinants of DR screening and this was mentioned in 47% of the studies [#1, #4, #7, #9, #11, #13, #14, #15, #16]. It was found that a shorter duration of Diabetes was frequently associated with defaulting on screening appointments. The effect of the type of Diabetes, family history of Diabetes, and the presence of comorbidities and history of previous eye disease were equally assessed in the studies.
- Health Beliefs: The influence of health beliefs underlying the perception of ill health and the need to seek care were equally explored. Studies [#5, #12, #17, #18] highlighted some of the factors such as stigma, fear of surgery, fear of vision loss and denial that could impact the choice of a person living with Diabetes to seek care. For example, in study #17 by Wentzel and Mchiza, fear of vision loss was cited by one of the key informants as a reason for seeking care and this outweighed fear of the surgical procedure. Commenting on this, one of the key informants interviewed stated “The main thing, I think they (patients) are scared”. Some of the patients also admitted their fear of treatment, by making remarks like “I was scared. I didn’t know what to expect” (Patient 8). “… if I don’t do it then I’ll eventually go blind” (Patient 3); “… probably my fear that I’ll go blind if I don’t go” (Patient 7).

### Ability to seek

- Demographic Factors: Seven studies [#4, #7, #8, #12, #13, #14, #16,] investigated the association between age and access of DRSS, with majority of the studies stating that age at onset was a significant predictor of DRSS access. While study #14 found strong evidence of association between having a dilated eye examination (ever) and increasing age, the effect size was quite small. Study #12, on the other hand, reported that age was not associated with the access of DRSS. Studies [#5, #16, #18] reported on the influence of gender on the access of Diabetic Retinopathy screening services with studies #5 and #18 finding that being male increased the likelihood of accessing DRSS. Four studies [#5, #7, #8, #9] examined the impact of the level of education and marital status on DR screening access while studies [#8, #9 and #14] looked at the effect of employment status and residence on DRSS access. Two studies [#2 and #5] quizzed respondents on the impact of cultural beliefs on screening access. While 53% of the respondents in study #2 reported that cultural and spiritual beliefs may be the reason why people did not access DRSS, it did not specify the specific cultural and spiritual beliefs that could have been responsible for this trend. Some study participants in study #5 cited the limited appreciation of the chronicity of Diabetes and a lack of understanding of the concept of being “sick” without symptoms as some cultural beliefs that led to poor DRSS access. Commenting on this, one respondent said, “Most Africans don’t have the habit of checking our health status until we get sick.”

### Ability to reach

- Residence, Mobility and Social Factors: Three of the included studies [#5, #12, #17] stressed the role of family, friends, and other forms of social support on the access of DR screening. The need for an escort to screening appointments and its attendant implications were highlighted by both patients [#5, #12] and service providers [#17]. The impact of current state of health and mobility on the access of available DR services was explored in studies [#7, #10, #16, #17]. In study #10, the current status of eye health was investigated and 50.8% of people living with Type 2 Diabetes in the study reporting they had no previous dilated eye examination because they had no eye problems/visual symptoms. Similarly, being in a poor state of health as a result of Diabetes or another disease was highlighted as a major barrier to the access of DRSS. Three studies [#8, #9, #14] reported on the influence of urban vs rural residence on access to DRSS, with living in urban areas reported as having a strong association with access of DRSS.

### Ability to pay

- Financial factors: Studies [#4, #5, #8, #13] explored the effect of different income levels and financial status on the access of DR screening services. Here, financial constraints and low income were reported as the reasons for the poor DRSS access. For example, in study #5, women were less likely to attend DR screening appointments because they did not have the financial independence to do so since in most families in the study, financial decisions were made by men. The availability of medical insurance coverage was equally highlighted as a predictor of access to DR screening facilities in studies [#5 and #7] while financial trade-offs and competing priorities were found to be significantly associated with the access of DRSS in studies [#12, #17, #18].

### Ability to engage

- Psychological Factors: Five studies [#9, #10, #12, #17, #18] explored the association between self-efficacy, attitude towards DR, anxiety and the access of DR screening services. In studies #12 and #18, lack of motivation, forgetfulness and a negative attitude towards DR were reported to be significantly associated with poor access of DR screening services (DRSS).

Service provider related themes were classified using the 5As service dimensions as follows:

### Approachability

- Service Reach: Three studies [#4, #8, #15] asked study participants about their source of information on Diabetes and diabetic eye disease, with majority of the study participants reporting healthcare providers as the main source of information. Other sources of information mentioned in the studies include friends and relatives, mass media and eyecare professionals. The lack of outreach services and mobile clinics was equally identified as a major obstacle to DR screening access in study #11.

### Acceptability

- Screening Competency: Three studies [#6, #11 and #19] asked healthcare providers about their experience levels in conducting DR screenings and how knowledgeable they are about Diabetic Retinopathy, most of the participants reported having good knowledge of diabetic ocular complications, however, it was also determined that only a few of the study participants routinely conducted retinal examinations on diabetic patients.

### Availability and accommodation

- Service Accessibility: Information on the accessibility of Diabetic Retinopathy screening service (DRSS) was solicited from service providers. Four studies [#3, #9, #10, #17] reported the ease of obtaining DR screening appointments as predictors of service utilisation while long waiting times was identified as a notable impediment in studies [#3, #17, #18]. Six studies [#2, #9, #10, #11, #12, #18] noted that travel distance to the screening service was a considerable predictor of DR screening access. Other factors indicated in the studies in relation to availability and accommodation include shortage of ophthalmic workforce [#9 and #19] and in study #17, the Covid19 pandemic was also mentioned as a remarkable cause of Diabetic Retinopathy screening service disruption during the lockdown period.

### Affordability

- Service Costs: The direct cost of treatment was mentioned as a notable determinant of Diabetic Retinopathy screening services access in 5 studies [#5, #7, #9, #10, #12]. Indirect expenses such as transport and accommodation costs were equally highlighted in study #5 and #12 while the mode of health service payment was considered as a significant factor in study #7.

### Appropriateness

- Service Coordination: Absence of functional screening equipment like ophthalmoscopes, fundus cameras, and diagnostic medications were highlighted as major hinderances to DR screening access in studies [#3, #6 and #11]. Task sharing between eye care professionals and the non-ophthalmic workforce were also identified as significant predictors of DR screening compliance in studies [#3, #9, #11, #17 and #19]. Some of the study participants in 37% of the studies reviewed [#1, #2, #4, #12, #14, #16, #17] reported there was poor access because their healthcare professional did not refer them to the Diabetic Retinopathy screening service (DRSS). Similarly, providing explanations on the outcome of retinal screening by healthcare professionals in studies [#3, #9, #17] and communication between services involved with the care of people living with diabetes were considered as factors affecting access of DRSS in studies #11 and #19 respectively.

**Fig 3:**
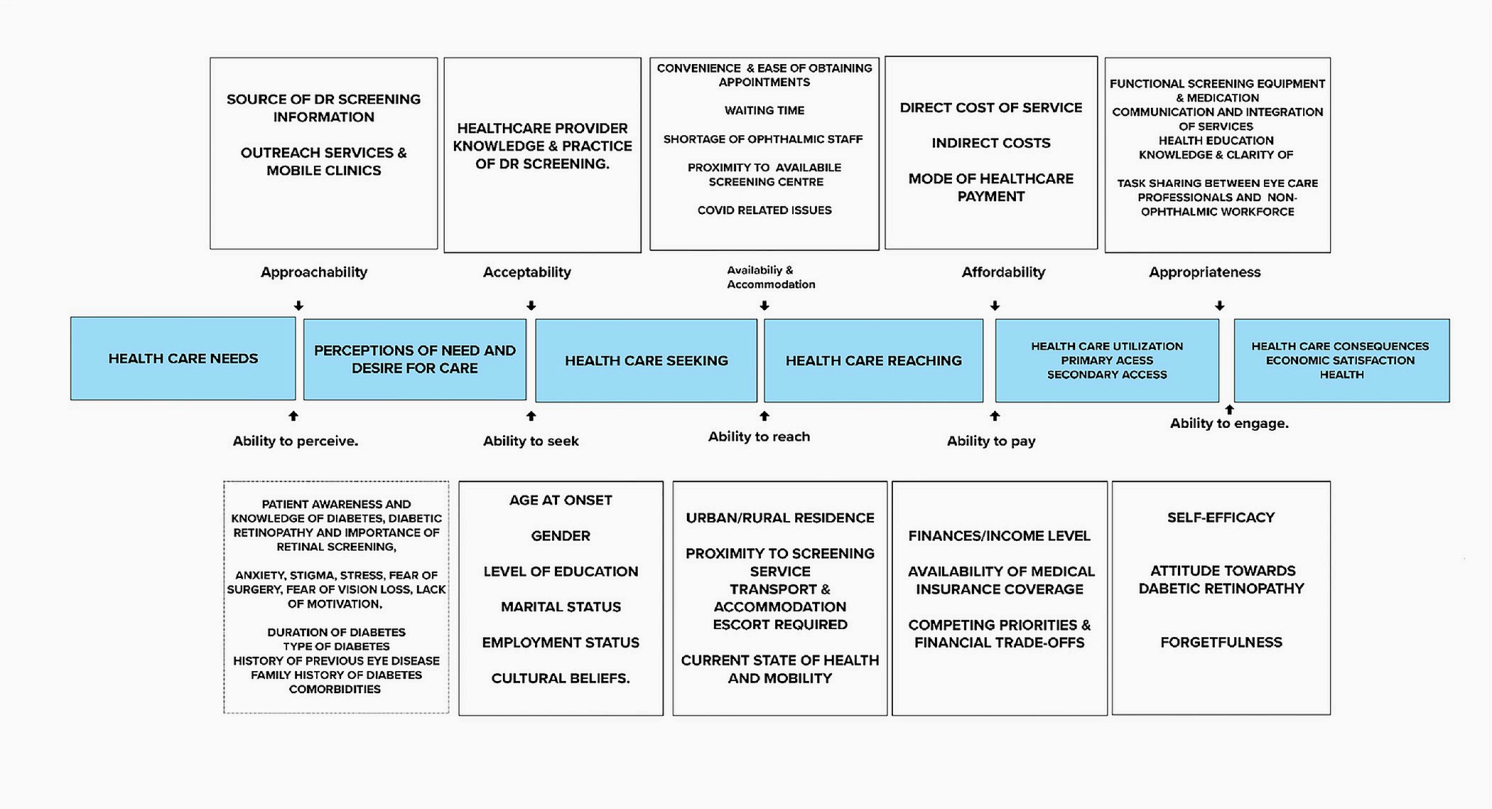
Mapping DR screening services using the 5As framework of access to healthcare.

The 5As framework was used to map these emerging themes as follows. See Table 2 and Fig 3.

**Table 2:** Thematic synthesis using the 5As framework of access to healthcare.

THEMES	5ADIMENSIONS AND ABILITIES	ARTICLES MENTIONED	%
PATIENT			
	**ABILITY TO PERCEIVE**	** **	
Health Literacy	Patient awareness and knowledge of Diabetes, Diabetic Retinopathy.	[#2, #4, #5, #8, #9, #10, #14, #13, #15, #16, #17, #18]	63
Health-related Factors	Duration of Diabetes	[#1, #4, #7, #9, #11, #13, #14, #15, #16]	47
	Type of Diabetes	[#4, #7, #8]	16
	History of previous eye Disease	[#1, #4, #8, #9, #16]	26
	Family history of Diabetes	[#9, #14, #17]	1
	Comorbidities	[#11, #14, #16, #17]	21
Health beliefs	Stigma, fear of surgery/ vision loss, denial	[#5, #12, #17, #18]	21
	**ABILITY TO SEEK**		
Demographic factors	Age at onset	[#4, #7, #8, #12, #13, #14, #16]	37
	Gender	[#5, #16, #18]	16
	Marital status	[#5, #7, #8, #9]	21
	Level of education	[#5, #7, #8, #9]	21
	Employment status	[#8, #9, #14]	16
	Cultural beliefs	[#2, #5]	11
	**ABILITY TO REACH**	** **	
Residence, Mobility & Social Factors	State of health and mobility	[#7, #10, #16, #17]	21
	Urban/Rural residence	[#8, #9, #14]	16
	Family & Social Support	[#5, #12, #17]	16
	**ABILITY TO PAY**		
Financial factors	Income Level	[#4, #5, #8, #13]	21
	Availability of medical insurance coverage	[#5, #7]	11
	Competing priorities & Financial trade-offs	[#12, #17, #18]	16
	**ABILITY TO ENGAGE**		
Psychological Factors	Self-Efficacy, Attitude towards Diabetic Retinopathy, Forgetfulness, Lack of Motivation, Anxiety	[#9, #10, #12, #17, #18]	26
**SERVICE**			
	**APPROACHABILITY**		
Service reach	Source of DR Screening information	[#4, #8, #15]	16
	Availability of outreach services	#11	5
	**ACCEPTABILITY**		
Screening competency	Healthcare provider knowledge and practice Of DR screening	[#6, #11, #19]	16
	**AVAILABILITY AND ACCOMMODATION**		
Service Accessibility	Convenience & Ease Of obtaining appointments	[#3, #9, #10, #17]	21
	Waiting time	[#3, #17, #18]	16
	Shortage of ophthalmic staff	[#9, #19]	11
	Proximity to available screening centers	[#2, #9, #10, #12, #18]	26
	COVID-19 related issues	#17	5
	**AFFORDABILITY**		
Service Costs	Direct cost of screening	[#5, #7, #9, #10, #12]	26
	Indirect cost of screening	[#5, #12]	11
	Mode of health service payment	#7	5
	**APPROPRIATENESS**		
Service Coordination	Functional screening equipment and diagnostic medication	[#3, #6, #11]	16
	Communication and Integration of services	[#11, #19]	11
	Health education by medical personnel	[3,9,17]	16
	Knowledge & clarity of referral protocol	[#1, #2, #4, #12, #14, #16, #17]	37
	Task sharing between eye care professionals and non-ophthalmic workforce	[#6, #11, #19]	16

5A dimensions/abilities are highlighted in blue rows, #-Study number, %- Percentage distribution of themes.

## 4 Discussion

This scoping review aimed to highlight the contextual factors associated with the access of DR screening services in line with the 5As conceptual framework of healthcare access. Health literacy and health-related factors like duration of Diabetes were noted as the most prominent factors associated with the access of DRSS. Knowledge and clarity of referral protocol as well as demographic factors such as the age of onset were equally highlighted as common factors.

### 4.1 Approachability/Ability to perceive

In terms of approachability of DR screening services in sub-Saharan Africa, most of the studies revealed screening was conducted mainly on an opportunistic basis since very few screening centres in the studies [#3 and #12] reported the existence of an electronic database for people living with Diabetes. This highlights remarkable infrastructural deficits in the health information systems needed for the invitation, recall and referral components of screening services [18]. Health care providers were cited as the main sources of information on regular eye checkup practices for people living with Diabetes in studies #4, #8 and #15 [35,39,46]. For service users, the ability to perceive is largely influenced by the level of knowledge and awareness possessed by an individual in relation to their health condition. Health beliefs like fear of blindness, stigma, and denial also play a vital role in the ability to perceive and seek care in these settings [41].Though regarded as a fundamental strategy used in improving health literacy, it was evident in a few of the studies that creating awareness of a disease condition alone does not necessarily guarantee attitudinal change that will lead to compliance. This was the case for both patients and service providers in studies #4 and #6 [35,37]. This finding tallies with a similar study in Indonesia which investigated how knowledge of DR improved screening practices among general practitioners [50]. Conversely, studies in Turkey, Hong Kong, and Saudi Arabia found significant associations between knowledge of DR and increased DR screening services access and treatment adherence [51–53]. This indicates that compliance to DR screening or treatment could be determined by a combination of factors with different variables playing a role in the process [54]. For instance, in study #2, some participants were non-compliant because they believed retinal screening would delay the process of acquiring new spectacles. This is because patients had to undergo retinal examinations first to ensure there were no abnormalities that would preclude prescribing of spectacles in that facility. This demonstrates how preconceived notions could impact compliance to screening [33]. Duration of Diabetes was frequently considered as a major predictor of Diabetic Retinopathy screening service (DRSS) access, with a shorter duration of Diabetes linked to poor compliance. This outcome was also resonated in a study carried out in Jeddah, Saudi Arabia[55] which found that duration of Diabetes and age were significant predictors of DR among people living with Diabetes.

### 4.2 Acceptability/Ability to seek

The rate of DR screening by eyecare practitioners and other healthcare providers in the reviewed studies was reportedly low as seen in studies #6, #11 and #19. Studies conducted in a high-income country like the United States of America also recorded a similar trend [56]. To ensure a screening service is acceptable, it is crucial to make sure the service is not only of high quality but culturally sensitive [57]. Demographic factors like age, gender, marital status, cultural beliefs and other social constructs have been shown to influence health seeking behaviour [58]. For instance, increasing age was noted as a major predictor of access to Diabetic Retinopathy screening services with a greater degree of non-compliance recorded in younger age groups[44,47]. This was also corroborated by a study in the United Kingdom which concluded that younger adults living with Diabetes had greater rates of screening non-attendance compared to older individuals [59]. In three of the studies [#5, #16, #18] that assessed the impact of gender on DR screening access, males had a higher rate of service utilisation in two studies, while study #16 found the contrary. The provision of gender sensitive interventions like the use of female health care workers to attend to diabetic women has been shown to improve screening compliance [60]. This review also revealed varying degrees of association between marital status and DR screening service access. In study #7, for instance, people who were divorced were more likely to utilise eye health services than their married counterparts [38]. Conversely, in a study of barriers to healthcare for women of different ethnic and racial backgrounds, it was shown that being single or divorced negatively impacted access to healthcare services [61].

### 4.3 Availability & Accommodation/Ability to reach

From the reviewed studies, we can deduce that where an individual resides and their proximity to healthcare facilities are important correlates of access to Diabetic Retinopathy screening services. People residing in urban or suburban settlements are more likely to gain quicker access to health information and tend to avail themselves of healthcare services more than their rural counterparts [62]. This also includes the availability of transportation, housing, and other forms of services to ensure optimal access to health services. The state of health and ambulation of a patient is also central to how they interact with the health system [63]. Some people might require the services of escorts to accompany them to their screening appointments and this could have far-reaching economic consequences for the parties involved, hence the presence of a robust social support system is considered a key determinant of screening adherence [36]. A study carried out in Egypt showed that lack of social support correlated positively with higher amounts of DR treatment drop-outs [64]. For service providers, availability entails the ease of booking screening appointments, waiting times and service convenience. The severe shortage of the eyecare workforce has been identified as a primary contributor to the inaccessibility of DR screening services [65]. Beyond recruiting additional human resources for eye health services, ensuring equitable distribution of staff across rural and urban areas is paramount [66]. Study #17 equally highlighted the service disruption brought about by unique circumstances such as the COVID-19 pandemic. This was true not just for DR screening services, but all other screening services regarded as non-essential services during the lockdown period [67].

### 4.4 Affordability/Ability to pay

Owing to limited resources and financial hardships that characterize sub–Saharan Africa, issues of direct and indirect cost of service, absence of medical insurance and competing priorities were mentioned as notable barriers to DRSS access. These prohibitive costs of care were equally highlighted as significant barriers to the timely access of DR screening services in a multi -country study to determine global perspectives on access to DR screening and treatment[68]. While the cost of DR screening and treatment is generally regarded as a major hinderance, it is noteworthy that some of the respondents in the reviewed studies did not consider DR screening a priority as seen in studies #10 and #12 respectively. This lack of prioritisation could influence willingness to pay for Diabetic Retinopathy screening services (DRSS) and this was investigated in a previous study in Taiwan [69]. In this Taiwanese study, it was found that level of income did not affect willingness to pay as much as level of education and degree of severity of Diabetic Retinopathy. Likewise, the availability of a robust medical health insurance scheme is considered an important predictor of DR screening and health service access [70]. Given the widespread poverty in sub-Saharan Africa, most people living with Diabetes ultimately rely on out-of-pocket expenses to keep up with their health needs leading to poor DRSS access as seen in study #5.

### 4.5 Appropriateness/Ability to engage

The introduction of innovative solutions and approaches like fundus photography, ocular coherence tomography, teleophthalmology and other novel means of improving screening efficiency has been hailed as major turning points in the trend of DR screening in resource-constrained settings [71]. Granted, these might go a long way to improve coverage in geographically inaccessible areas, however, the training of non-physician clinical officers and nurses on how to use these devices and interpret retinal photographs to accurately identify true cases of vision-threatening Diabetic Retinopathy (VTDR) is also needed [72]. These task-sharing strategies, when implemented at the primary care level could cushion the enormous workload on a terribly understaffed eye care sector region-wide [73]. Even though this is generally a capital-intensive venture with a huge potential to yield promising return on investments, the cost of maintenance and other overhead cost incurred over time are also worth-considering [74].

Finally, the absence of clear referral protocols to ophthalmologists by general practitioners or other qualified healthcare practitioners was highlighted as an impediment to the timely access of DRSS in studies [#1, #2, #4, #12, #14, #16, #17]. This could possibly emanate from insufficient knowledge on how to grade different stages of DR or lack of an existing referral pathway. Similarly, a systematic review involving a couple of low-and-middle-income countries revealed a national DR screening protocol is non-existent in most sub-Saharan African countries [2]. Furthermore, knowledge of the referral pathway alone does not suffice, the need for stringent follow-up care cannot be overemphasized [75]. Efforts to keep the channels of communication between the diabetic clinics and ophthalmology departments open is paramount [42]. There is need to develop integrated models of care for all facilities involved in the care and management of people living with Diabetes [76]. In addition, incorporating a system of constant DR screening reminders through mass media campaigns and personalized phone reminders could encourage people living with Diabetes to seek timely care [77]. Considering that people with vision-threatening Diabetic Retinopathy are often asymptomatic, committing to regular Diabetic Retinopathy screening using appropriate technology and adequately trained personnel could potentially ensure timely interventions culminating in better visual outcomes for people living with Diabetes [78,79].

## 5 Study limitations

Owing to time constraints, this study did not include a consultation phase as recommended by Levac et al [80]. Some studies highlighted cultural beliefs as reasons for poor access without mentioning the specific cultural practices that could impact DR screening access. In addition, some of the studies required respondents to recall events that happened in the past, which makes their responses subject to recall bias. The 5As framework contained a few overlapping themes which might have resulted in some analytical ambiguity. There was also language bias since only articles published in English were included.

## 6 Conclusion and recommendation

This review highlighted the contextual challenges and facilitators of access to DR screening. While the establishment of effective screening models to monitor and initiate timely intervention is vital, understanding the issues surrounding the access of these services will help policymakers and other stakeholders develop a system that promotes integration, communication, and compliance. The 5As framework of access to healthcare provides a holistic view of factors associated with the access of DR screening services through the lenses of patients and service providers. It portrays access as a dynamic process rather than a static concept. Key factors impacting access to DR screening services in this review include health literacy, duration of Diabetes, age at onset, and insufficient referrals by healthcare providers, which correspond to the 5As dimensions of ability to perceive, ability to seek, and appropriateness. There is need for further research on modalities to strengthen health systems especially in areas of human resources for eye health, healthcare financing and integration of eye care services to address these highlighted contextual barriers of DR screening and facilitate the provision and optimization of DR screening services in sub-Saharan Africa.

## Supporting information

S1 Table Search strategy and search histories. (DOCX)

S1 Appendix Scoping review protocol. (DOCX)

S2 Appendix PRISMA checklist. (DOCX)
