# Supplementary material for: Factors associated with the uptake and utilisation of diabetic retinopathy screening services in sub-Saharan Africa: A scoping review
Source: PLoS One. 2024 Dec 13;19(12):e0315367. doi: 10.1371/journal.pone.0315367 (PMC11643260; doi:10.1371/journal.pone.0315367)
Supplement: S1 Table — (DOCX) [file pone.0315367.s001.docx]

# Search strategy and search histories applied to databases (PubMed and CINAHL Complete

| **Title of the Research** | **SCOPING REVIEW OF THE FACTORS ASSOCIATED WITH THE UPTAKE AND UTILIZATION OF DIABETIC RETINOPATHY SCREENING SERVICES IN SUB-SAHARAN AFRICA** |
| --- | --- |
| **Databases** | PubMed, Medline, Embase, APA PsycINFO, Web of Science, CINHAL Complete, Africa Journals Online. |
| **Date** | N/A |
| **Framework (PCC)** | Population: People living with Diabetes  Concept: Uptake and Utilization of Diabetic Retinopathy screening services  Context: sub-Saharan Africa |
| **Diabetic Retinopathy** | *“Diabetes eye disease” OR “Diabetic Retinopathy” OR “Diabetic macula oedema” OR “Diabetic macula edema” OR “Diabetic maculopathy”* |
| **Screening** | *“Screening Programme*” OR “Screening services” OR “Diabetic Eye examinations” OR “Diabetic retinal examinations”* *OR* *Screening OR “systematic screening” OR “mass screening” OR “opportunistic screening” OR* “vision screening” OR “eye test” OR “Vision test” OR “Eye Assessment” OR “Vision Assessment” OR “retinal imaging” OR “slit lamp biomicroscopy” OR ophthalmoscopy OR “fundus photography” OR “Ocular coherence Tomography” |
| **Sub-Saharan Africa** | **‘****’sub Saharan Africa’’ OR ‘’sub-Saharan Africa” OR Africa OR Angola OR Benin or Botswana ‘’Burkina Faso’’ OR Burundi OR Cameroon OR ‘’Central Africa Republic’’ OR "Cape Verde" OR Chad OR Comoros OR ‘’Cote d’Ivoire’’ OR Djibouti OR ‘’Democratic Republic of the Congo’’ OR Eritrea OR Eswatini OR ‘’Equatorial Guinea" OR Ethiopia OR Gabon OR Ghana OR Guinea OR "Guinea-Bissau" OR Kenya OR Lesotho OR Liberia OR Madagascar OR Malawi OR Mali OR Mauritius OR Mauritania OR Mozambique OR Namibia OR Niger OR Nigeria OR ‘’Republic of the Congo’’ OR Rwanda OR ‘’Sao Tome and Principe’’ OR Senegal OR Seychelles OR "Sierra Leone" OR Somalia OR "South Africa" OR "South Sudan" OR Sudan OR Tanzania OR Togo OR Uganda OR Zambia OR Zimbabwe** |

**Search history from PubMed**

| **Search number** | **Query** | **Search Details** | **Results** |
| --- | --- | --- | --- |
| **5** | **#3 AND #4** | **((((("africa south of the sahara"[MeSH Terms] OR ("africa"[All Fields] AND "south"[All Fields] AND "sahara"[All Fields]) OR "africa south of the sahara"[All Fields] OR ("sub"[All Fields] AND "saharan"[All Fields] AND "africa"[All Fields]) OR "sub saharan africa"[All Fields] OR ("africa south of the sahara"[MeSH Terms] OR ("africa"[All Fields] AND "south"[All Fields] AND "sahara"[All Fields]) OR "africa south of the sahara"[All Fields] OR ("sub"[All Fields] AND "saharan"[All Fields] AND "africa"[All Fields]) OR "sub saharan africa"[All Fields])) AND ("africa"[MeSH Terms] OR "africa"[All Fields] OR "africa s"[All Fields] OR "africas"[All Fields] OR ("angola"[MeSH Terms] OR "angola"[All Fields] OR "angola s"[All Fields]) OR ("benin"[MeSH Terms] OR "benin"[All Fields] OR "benin s"[All Fields]) OR (("botswana"[MeSH Terms] OR "botswana"[All Fields] OR "botswana s"[All Fields]) AND ("burkina faso"[MeSH Terms] OR ("burkina"[All Fields] AND "faso"[All Fields]) OR "burkina faso"[All Fields])) OR ("burundi"[MeSH Terms] OR "burundi"[All Fields]) OR ("cameroon"[MeSH Terms] OR "cameroon"[All Fields] OR "cameroons"[All Fields] OR "cameroon s"[All Fields]) OR (("africa, central"[MeSH Terms] OR ("africa"[All Fields] AND "central"[All Fields]) OR "central africa"[All Fields] OR ("central"[All Fields] AND "africa"[All Fields])) AND ("republic"[All Fields] OR "republic s"[All Fields] OR "republics"[All Fields])))) AND ("cabo verde"[MeSH Terms] OR ("cabo"[All Fields] AND "verde"[All Fields]) OR "cabo verde"[All Fields] OR ("cape"[All Fields] AND "verde"[All Fields]) OR "cape verde"[All Fields])) AND ("chad"[MeSH Terms] OR "chad"[All Fields] OR ("comoros"[MeSH Terms] OR "comoros"[All Fields] OR "comoro"[All Fields]) OR ("cote d ivoire"[MeSH Terms] OR ("cote"[All Fields] AND "d ivoire"[All Fields]) OR "cote d ivoire"[All Fields]) OR ("djibouti"[MeSH Terms] OR "djibouti"[All Fields]) OR ("democratic republic of the congo"[MeSH Terms] OR ("democratic"[All Fields] AND "republic"[All Fields] AND "congo"[All Fields]) OR "democratic republic of the congo"[All Fields]) OR ("eritrea"[MeSH Terms] OR "eritrea"[All Fields]) OR ("eswatini"[MeSH Terms] OR "eswatini"[All Fields]) OR ("equatorial guinea"[MeSH Terms] OR ("equatorial"[All Fields] AND "guinea"[All Fields]) OR "equatorial guinea"[All Fields]))) OR ("ethiopia"[MeSH Terms] OR "ethiopia"[All Fields] OR "ethiopia s"[All Fields]) OR ("gabon"[MeSH Terms] OR "gabon"[All Fields]) OR ("ghana"[MeSH Terms] OR "ghana"[All Fields] OR "ghana s"[All Fields]) OR ("guinea"[MeSH Terms] OR "guinea"[All Fields] OR "guinea s"[All Fields] OR "guineas"[All Fields]) OR "Guinea-Bissau"[All Fields] OR ("kenya"[MeSH Terms] OR "kenya"[All Fields] OR "kenya s"[All Fields]) OR ("lesotho"[MeSH Terms] OR "lesotho"[All Fields]) OR ("liberia"[MeSH Terms] OR "liberia"[All Fields] OR "liberia s"[All Fields]) OR ("madagascar"[MeSH Terms] OR "madagascar"[All Fields] OR "madagascar s"[All Fields]) OR ("malawi"[MeSH Terms] OR "malawi"[All Fields] OR "malawi s"[All Fields]) OR ("mali"[MeSH Terms] OR "mali"[All Fields]) OR ("mauritius"[MeSH Terms] OR "mauritius"[All Fields]) OR ("mauritania"[MeSH Terms] OR "mauritania"[All Fields]) OR ("mozambique"[MeSH Terms] OR "mozambique"[All Fields] OR "mozambique s"[All Fields]) OR ("namibia"[MeSH Terms] OR "namibia"[All Fields] OR "namibia s"[All Fields]) OR ("niger"[MeSH Terms] OR "niger"[All Fields]) OR ("nigeria"[MeSH Terms] OR "nigeria"[All Fields] OR "nigeria s"[All Fields]) OR ("congo"[MeSH Terms] OR "congo"[All Fields] OR ("republic"[All Fields] AND "congo"[All Fields]) OR "republic of the congo"[All Fields]) OR ("rwanda"[MeSH Terms] OR "rwanda"[All Fields] OR "rwanda s"[All Fields]) OR ("sao tome and principe"[MeSH Terms] OR ("sao"[All Fields] AND "tome"[All Fields] AND "principe"[All Fields]) OR "sao tome and principe"[All Fields]) OR ("senegal"[MeSH Terms] OR "senegal"[All Fields] OR "senegal s"[All Fields]) OR ("seychelles"[MeSH Terms] OR "seychelles"[All Fields]) OR "Sierra Leone"[All Fields] OR ("somalia"[MeSH Terms] OR "somalia"[All Fields] OR "somalia s"[All Fields]) OR "South Africa"[All Fields] OR "South Sudan"[All Fields] OR ("sudan"[MeSH Terms] OR "sudan"[All Fields] OR "sudans"[All Fields] OR "sudan s"[All Fields]) OR ("tanzania"[MeSH Terms] OR "tanzania"[All Fields] OR "tanzania s"[All Fields]) OR ("togo"[MeSH Terms] OR "togo"[All Fields]) OR ("uganda"[MeSH Terms] OR "uganda"[All Fields] OR "uganda s"[All Fields]) OR ("zambia"[MeSH Terms] OR "zambia"[All Fields] OR "zambia s"[All Fields]) OR ("zimbabwe"[MeSH Terms] OR "zimbabwe"[All Fields] OR "zimbabwe s"[All Fields])) AND (("Diabetes eye disease"[All Fields] OR "Diabetic Retinopathy"[All Fields] OR "Diabetic macula oedema"[All Fields] OR "Diabetic macula edema"[All Fields] OR "Diabetic maculopathy"[All Fields]) AND ("screening programme*"[All Fields] OR "Screening services"[All Fields] OR "Diabetic Eye examinations"[All Fields] OR "Diabetic retinal examinations"[All Fields] OR ("diagnosis"[MeSH Subheading] OR "diagnosis"[All Fields] OR "screening"[All Fields] OR "mass screening"[MeSH Terms] OR ("mass"[All Fields] AND "screening"[All Fields]) OR "mass screening"[All Fields] OR "early detection of cancer"[MeSH Terms] OR ("early"[All Fields] AND "detection"[All Fields] AND "cancer"[All Fields]) OR "early detection of cancer"[All Fields] OR "screen"[All Fields] OR "screenings"[All Fields] OR "screened"[All Fields] OR "screens"[All Fields]) OR "systematic screening"[All Fields] OR "mass screening"[All Fields] OR "opportunistic screening"[All Fields] OR "vision screening"[All Fields] OR "eye test"[All Fields] OR "Vision test"[All Fields] OR "Eye Assessment"[All Fields] OR "Vision Assessment"[All Fields] OR "retinal imaging"[All Fields] OR "slit lamp biomicroscopy"[All Fields] OR ("ophthalmoscopy"[MeSH Terms] OR "ophthalmoscopy"[All Fields] OR "ophthalmoscopies"[All Fields]) OR "fundus photography"[All Fields] OR "Ocular coherence Tomography"[All Fields]))** | **198** |
| **4** | **#1 AND #2** | **("Diabetes eye disease"[All Fields] OR "Diabetic Retinopathy"[All Fields] OR "Diabetic macula oedema"[All Fields] OR "Diabetic macula edema"[All Fields] OR "Diabetic maculopathy"[All Fields]) AND ("screening programme*"[All Fields] OR "Screening services"[All Fields] OR "Diabetic Eye examinations"[All Fields] OR "Diabetic retinal examinations"[All Fields] OR ("diagnosis"[MeSH Subheading] OR "diagnosis"[All Fields] OR "screening"[All Fields] OR "mass screening"[MeSH Terms] OR ("mass"[All Fields] AND "screening"[All Fields]) OR "mass screening"[All Fields] OR "early detection of cancer"[MeSH Terms] OR ("early"[All Fields] AND "detection"[All Fields] AND "cancer"[All Fields]) OR "early detection of cancer"[All Fields] OR "screen"[All Fields] OR "screenings"[All Fields] OR "screened"[All Fields] OR "screens"[All Fields]) OR "systematic screening"[All Fields] OR "mass screening"[All Fields] OR "opportunistic screening"[All Fields] OR "vision screening"[All Fields] OR "eye test"[All Fields] OR "Vision test"[All Fields] OR "Eye Assessment"[All Fields] OR "Vision Assessment"[All Fields] OR "retinal imaging"[All Fields] OR "slit lamp biomicroscopy"[All Fields] OR ("ophthalmoscopy"[MeSH Terms] OR "ophthalmoscopy"[All Fields] OR "ophthalmoscopies"[All Fields]) OR "fundus photography"[All Fields] OR "Ocular coherence Tomography"[All Fields])** | **14,529** |
| **3** | **‘’sub Saharan Africa’’ OR ‘’sub-Saharan Africa" OR Africa OR Angola OR Benin or Botswana ‘’Burkina Faso’’ OR Burundi OR Cameroon OR ‘’Central Africa Republic’’ OR "Cape Verde" OR Chad OR Comoros OR ‘’Cote d’Ivoire’’ OR Djibouti OR ‘’Democratic Republic of the Congo’’ OR Eritrea OR Eswatini OR ‘’Equatorial Guinea" OR Ethiopia OR Gabon OR Ghana OR Guinea OR "Guinea-Bissau" OR Kenya OR Lesotho OR Liberia OR Madagascar OR Malawi OR Mali OR Mauritius OR Mauritania OR Mozambique OR Namibia OR Niger OR Nigeria OR ‘’Republic of the Congo’’ OR Rwanda OR ‘’Sao Tome and Principe’’ OR Senegal OR Seychelles OR "Sierra Leone" OR Somalia OR "South Africa" OR "South Sudan" OR Sudan OR Tanzania OR Togo OR Uganda OR Zambia OR Zimbabwe** | **(((("africa south of the sahara"[MeSH Terms] OR ("africa"[All Fields] AND "south"[All Fields] AND "sahara"[All Fields]) OR "africa south of the sahara"[All Fields] OR ("sub"[All Fields] AND "saharan"[All Fields] AND "africa"[All Fields]) OR "sub saharan africa"[All Fields] OR ("africa south of the sahara"[MeSH Terms] OR ("africa"[All Fields] AND "south"[All Fields] AND "sahara"[All Fields]) OR "africa south of the sahara"[All Fields] OR ("sub"[All Fields] AND "saharan"[All Fields] AND "africa"[All Fields]) OR "sub saharan africa"[All Fields])) AND ("africa"[MeSH Terms] OR "africa"[All Fields] OR "africa s"[All Fields] OR "africas"[All Fields] OR ("angola"[MeSH Terms] OR "angola"[All Fields] OR "angola s"[All Fields]) OR ("benin"[MeSH Terms] OR "benin"[All Fields] OR "benin s"[All Fields]) OR (("botswana"[MeSH Terms] OR "botswana"[All Fields] OR "botswana s"[All Fields]) AND ("burkina faso"[MeSH Terms] OR ("burkina"[All Fields] AND "faso"[All Fields]) OR "burkina faso"[All Fields])) OR ("burundi"[MeSH Terms] OR "burundi"[All Fields]) OR ("cameroon"[MeSH Terms] OR "cameroon"[All Fields] OR "cameroons"[All Fields] OR "cameroon s"[All Fields]) OR (("africa, central"[MeSH Terms] OR ("africa"[All Fields] AND "central"[All Fields]) OR "central africa"[All Fields] OR ("central"[All Fields] AND "africa"[All Fields])) AND ("republic"[All Fields] OR "republic s"[All Fields] OR "republics"[All Fields])))) AND ("cabo verde"[MeSH Terms] OR ("cabo"[All Fields] AND "verde"[All Fields]) OR "cabo verde"[All Fields] OR ("cape"[All Fields] AND "verde"[All Fields]) OR "cape verde"[All Fields])) AND ("chad"[MeSH Terms] OR "chad"[All Fields] OR ("comoros"[MeSH Terms] OR "comoros"[All Fields] OR "comoro"[All Fields]) OR ("cote d ivoire"[MeSH Terms] OR ("cote"[All Fields] AND "d ivoire"[All Fields]) OR "cote d ivoire"[All Fields]) OR ("djibouti"[MeSH Terms] OR "djibouti"[All Fields]) OR ("democratic republic of the congo"[MeSH Terms] OR ("democratic"[All Fields] AND "republic"[All Fields] AND "congo"[All Fields]) OR "democratic republic of the congo"[All Fields]) OR ("eritrea"[MeSH Terms] OR "eritrea"[All Fields]) OR ("eswatini"[MeSH Terms] OR "eswatini"[All Fields]) OR ("equatorial guinea"[MeSH Terms] OR ("equatorial"[All Fields] AND "guinea"[All Fields]) OR "equatorial guinea"[All Fields]))) OR ("ethiopia"[MeSH Terms] OR "ethiopia"[All Fields] OR "ethiopia s"[All Fields]) OR ("gabon"[MeSH Terms] OR "gabon"[All Fields]) OR ("ghana"[MeSH Terms] OR "ghana"[All Fields] OR "ghana s"[All Fields]) OR ("guinea"[MeSH Terms] OR "guinea"[All Fields] OR "guinea s"[All Fields] OR "guineas"[All Fields]) OR "Guinea-Bissau"[All Fields] OR ("kenya"[MeSH Terms] OR "kenya"[All Fields] OR "kenya s"[All Fields]) OR ("lesotho"[MeSH Terms] OR "lesotho"[All Fields]) OR ("liberia"[MeSH Terms] OR "liberia"[All Fields] OR "liberia s"[All Fields]) OR ("madagascar"[MeSH Terms] OR "madagascar"[All Fields] OR "madagascar s"[All Fields]) OR ("malawi"[MeSH Terms] OR "malawi"[All Fields] OR "malawi s"[All Fields]) OR ("mali"[MeSH Terms] OR "mali"[All Fields]) OR ("mauritius"[MeSH Terms] OR "mauritius"[All Fields]) OR ("mauritania"[MeSH Terms] OR "mauritania"[All Fields]) OR ("mozambique"[MeSH Terms] OR "mozambique"[All Fields] OR "mozambique s"[All Fields]) OR ("namibia"[MeSH Terms] OR "namibia"[All Fields] OR "namibia s"[All Fields]) OR ("niger"[MeSH Terms] OR "niger"[All Fields]) OR ("nigeria"[MeSH Terms] OR "nigeria"[All Fields] OR "nigeria s"[All Fields]) OR ("congo"[MeSH Terms] OR "congo"[All Fields] OR ("republic"[All Fields] AND "congo"[All Fields]) OR "republic of the congo"[All Fields]) OR ("rwanda"[MeSH Terms] OR "rwanda"[All Fields] OR "rwanda s"[All Fields]) OR ("sao tome and principe"[MeSH Terms] OR ("sao"[All Fields] AND "tome"[All Fields] AND "principe"[All Fields]) OR "sao tome and principe"[All Fields]) OR ("senegal"[MeSH Terms] OR "senegal"[All Fields] OR "senegal s"[All Fields]) OR ("seychelles"[MeSH Terms] OR "seychelles"[All Fields]) OR "Sierra Leone"[All Fields] OR ("somalia"[MeSH Terms] OR "somalia"[All Fields] OR "somalia s"[All Fields]) OR "South Africa"[All Fields] OR "South Sudan"[All Fields] OR ("sudan"[MeSH Terms] OR "sudan"[All Fields] OR "sudans"[All Fields] OR "sudan s"[All Fields]) OR ("tanzania"[MeSH Terms] OR "tanzania"[All Fields] OR "tanzania s"[All Fields]) OR ("togo"[MeSH Terms] OR "togo"[All Fields]) OR ("uganda"[MeSH Terms] OR "uganda"[All Fields] OR "uganda s"[All Fields]) OR ("zambia"[MeSH Terms] OR "zambia"[All Fields] OR "zambia s"[All Fields]) OR ("zimbabwe"[MeSH Terms] OR "zimbabwe"[All Fields] OR "zimbabwe s"[All Fields])** | **635,615** |
| **2** | **"Screening Programme*" OR "Screening services" OR "Diabetic Eye examinations" OR "Diabetic retinal examinations" OR Screening OR "systematic screening" OR "mass screening" OR "opportunistic screening" OR "vision screening" OR "eye test" OR "Vision test" OR "Eye Assessment" OR "Vision Assessment" OR "retinal imaging" OR "slit lamp biomicroscopy" OR ophthalmoscopy OR "fundus photography" OR "Ocular coherence Tomography"** | **"screening programme*"[All Fields] OR "Screening services"[All Fields] OR "Diabetic Eye examinations"[All Fields] OR "Diabetic retinal examinations"[All Fields] OR ("diagnosis"[MeSH Subheading] OR "diagnosis"[All Fields] OR "screening"[All Fields] OR "mass screening"[MeSH Terms] OR ("mass"[All Fields] AND "screening"[All Fields]) OR "mass screening"[All Fields] OR "early detection of cancer"[MeSH Terms] OR ("early"[All Fields] AND "detection"[All Fields] AND "cancer"[All Fields]) OR "early detection of cancer"[All Fields] OR "screen"[All Fields] OR "screenings"[All Fields] OR "screened"[All Fields] OR "screens"[All Fields]) OR "systematic screening"[All Fields] OR "mass screening"[All Fields] OR "opportunistic screening"[All Fields] OR "vision screening"[All Fields] OR "eye test"[All Fields] OR "Vision test"[All Fields] OR "Eye Assessment"[All Fields] OR "Vision Assessment"[All Fields] OR "retinal imaging"[All Fields] OR "slit lamp biomicroscopy"[All Fields] OR ("ophthalmoscopy"[MeSH Terms] OR "ophthalmoscopy"[All Fields] OR "ophthalmoscopies"[All Fields]) OR "fundus photography"[All Fields] OR "Ocular coherence Tomography"[All Fields]** | **5,978,716** |
| **1** | **"Diabetes eye disease" OR "Diabetic Retinopathy" OR "Diabetic macula oedema" OR "Diabetic macula edema" OR "Diabetic maculopathy"** | **"Diabetes eye disease"[All Fields] OR "Diabetic Retinopathy"[All Fields] OR "Diabetic macula oedema"[All Fields] OR "Diabetic macula edema"[All Fields] OR "Diabetic maculopathy"[All Fields]** | **40,895** |

Top of Form

[Update My Account](https://web.p.ebscohost.com/ehost/Toolbar/update?sid=16134b02-b1c5-4993-b4db-c0a3fe35cedf@redis&vid=16&ReturnUrl=%252fehost%252falert%253fvid%253d16%2526sid%253d16134b02-b1c5-4993-b4db-c0a3fe35cedf%252540redis) Not IHEANYI ? [Sign in here.](https://web.p.ebscohost.com/ehost/Toolbar/SignInHere?sid=16134b02-b1c5-4993-b4db-c0a3fe35cedf@redis&vid=16&ReturnUrl=%252fehost%252falert%253fvid%253d16%2526sid%253d16134b02-b1c5-4993-b4db-c0a3fe35cedf%252540redis)

- [New Search](https://web.p.ebscohost.com/ehost/Toolbar/DoRedirect?sid=16134b02-b1c5-4993-b4db-c0a3fe35cedf@redis&vid=16&theDb=&theContentType=)
- [MeSH 2023](https://web.p.ebscohost.com/ehost/Toolbar/DoRedirect?sid=16134b02-b1c5-4993-b4db-c0a3fe35cedf@redis&vid=16&theDb=medmesh&theContentType=MESH)
- [Publications](https://web.p.ebscohost.com/ehost/Toolbar/DoRedirect?sid=16134b02-b1c5-4993-b4db-c0a3fe35cedf@redis&vid=16&theDb=mnhjnh&theContentType=Hierarchical+Journal)
- [Images](https://web.p.ebscohost.com/ehost/Toolbar/DoRedirect?sid=16134b02-b1c5-4993-b4db-c0a3fe35cedf@redis&vid=16&theDb=iqv&theContentType=Multimedia)
- [Indexes](https://web.p.ebscohost.com/ehost/Toolbar/DoRedirect?sid=16134b02-b1c5-4993-b4db-c0a3fe35cedf@redis&vid=16&theDb=&theContentType=Indexes)

- [Sign Out](https://web.p.ebscohost.com/ehost/Toolbar/SignInOut?sid=16134b02-b1c5-4993-b4db-c0a3fe35cedf@redis&vid=16)
- [Folder](https://web.p.ebscohost.com/ehost/Toolbar/OnRedirectLinkClick?sid=16134b02-b1c5-4993-b4db-c0a3fe35cedf@redis&vid=16&theTarget=folder&ReturnUrl=%252fehost%252falert%253fvid%253d16%2526sid%253d16134b02-b1c5-4993-b4db-c0a3fe35cedf%252540redis)
- [Preferences](javascript:void(0))
- [Languages](javascript:void(0))
- Help[Help](javascript:openWideTip('https://support.ebsco.com/help/?int=ehost&lang=en&feature_id=&TOC_ID=Always&SI=0&BU=0&GU=1&PS=0&ver=&dbs=mnh%27))

[**Accessibility Information and Tips**](javascript:openWideTip('https://support.ebsco.com/help/?int=ehost&lang=en&feature_id=access&TOC_ID=Always&SI=0&BU=0&GU=1&PS=0&ver=&dbs=mnh%27))

**Create or Edit Saved Searches**

[**UNIVERSITY OF LIMERICK GLUCKSMAN LIBRARY**](https://www.ul.ie/library)

IHEANYI 's Folder [
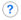
](javascript:openWideTip('https://support.ebsco.com/help/?int=ehost&lang=en&feature_id=custfolder&TOC_ID=Always&SI=0&BU=0&GU=1&PS=0&ver=live&dbs=%27))

[Back](javascript:__doPostBack('ctl00$ctl00$FindField$FindField$folderPageHeader$btnBack$lnkBack',''))

**Folder List**

| \| Name of Search/Alert \|  \| \| --- \| --- \| \| Description \|  \| \| Date Created \| 6/24/2023 \| \| Databases \|  \| \| Search Strategy \| ( ‘’sub Saharan Africa’’ OR ‘’sub-Saharan Africa” OR Africa OR Angola OR Benin or Botswana ‘’Burkina Faso’’ OR Burundi OR Cameroon OR ‘’Central Africa Republic’’ OR "Cape Verde" OR Chad OR Comoros OR ‘’Cote d’Ivoire’’ OR Djibouti OR ‘’Democratic Republic of the Congo’’ OR Eritrea OR Eswatini OR ‘’Equatorial Guinea" OR Ethiopia OR Gabon OR Ghana OR Guinea OR "Guinea-Bissau" OR Kenya OR Lesotho OR Liberia OR Madagascar OR Malawi OR Mali OR Mauritius OR Mauritania OR Mozambique OR Namibia OR Niger OR Nigeria OR ‘’Republic of the Congo’’ OR Rwanda OR ‘’Sao Tome and Principe’’ OR Senegal OR Seychelles OR "Sierra Leone" OR Somalia OR "South Africa" OR "South Sudan" OR Sudan OR Tanzania OR Togo OR Uganda OR Zambia OR Zimbabwe. ) AND ( “Screening Programme*” OR “Screening services” OR “Diabetic Eye examinations” OR “Diabetic retinal examinations” OR Screening OR “systematic screening” OR “mass screening” OR “opportunistic screening” OR “vision screening” OR “eye test” OR “Vision test” OR “Eye Assessment” OR “Vision Assessment” OR “retinal imaging” OR “slit lamp biomicroscopy” OR ophthalmoscopy OR “fundus photography” OR “Ocular coherence Tomography” ) AND ( “Diabetic eye disease” OR “Diabetic Retinopathy” OR “Diabetic macula oedema” OR “Diabetic macula edema” OR “Diabetic maculopathy” ) \| \| Interface \| EBSCOhost \| \| Save Search As \| \| Saved Search (Permanent) \| \| --- \| \| Saved Search (Temporary, 24 hours) \| \| Alert \| \| |
| --- | --- | --- | --- | --- | --- | --- | --- | --- | --- | --- | --- | --- | --- | --- | --- | --- | --- |
|     |

**Search History**

| \| **#** \| **Query** \| **Limiters/Expanders** \| **Last Run Via** \| **Results** \| **Action** \| \| --- \| --- \| --- \| --- \| --- \| --- \| \| S4 \| ( ‘’sub Saharan Africa’’ OR ‘’sub-Saharan Africa” OR Africa OR Angola OR Benin or Botswana ‘’Burkina Faso’’ OR Burundi OR Cameroon OR ‘’Central Africa Republic’’ OR "Cape Verde" OR Chad OR Comoros OR ‘’Cote d’Ivoire’’ OR Djibouti OR ‘’Democratic Republic of the Congo’’ OR Eritrea OR Eswatini OR ‘’Equatorial Guinea" OR Ethiopia OR Gabon OR Ghana OR Guinea OR "Guinea-Bissau" OR Kenya OR Lesotho OR Liberia OR Madagascar OR Malawi OR Mali OR Mauritius OR Mauritania OR Mozambique OR Namibia OR Niger OR Nigeria OR ‘’Republic of the Congo’’ OR Rwanda OR ‘’Sao Tome and Principe’’ OR Senegal OR Seychelles OR "Sierra Leone" OR Somalia OR "South Africa" OR "South Sudan" OR Sudan OR Tanzania OR Togo OR Uganda OR Zambia OR Zimbabwe. ) AND ( “Screening Programme*” OR “Screening services” OR “Diabetic Eye examinations” OR “Diabetic retinal examinations” OR Screening OR “systematic screening” OR “mass screening” OR “opportunistic screening” OR “vision screening” OR “eye test” OR “Vision test” OR “Eye Assessment” OR “Vision Assessment” OR “retinal imaging” OR “slit lamp biomicroscopy” OR ophthalmoscopy OR “fundus photography” OR “Ocular coherence Tomography” ) AND ( “Diabetic eye disease” OR “Diabetic Retinopathy” OR “Diabetic macula oedema” OR “Diabetic macula edema” OR “Diabetic maculopathy” ) \| Expanders - Apply equivalent subjects Search modes - Boolean/Phrase \| Interface - EBSCOhost Research Databases Search Screen - Advanced Search Database - CINAHL Complete \| 23 \| [Edit](javascript:__doPostBack('ctl00$ctl00$MainContentArea$MainContentArea$editControl$printHistory$HistoryRepeater$ctl00$linkEditSearch',''))S4 \| \| S3 \| ( ‘’sub Saharan Africa’’ OR ‘’sub-Saharan Africa” OR Africa OR Angola OR Benin or Botswana ‘’Burkina Faso’’ OR Burundi OR Cameroon OR ‘’Central Africa Republic’’ OR "Cape Verde" OR Chad OR Comoros OR ‘’Cote d’Ivoire’’ OR Djibouti OR ‘’Democratic Republic of the Congo’’ OR Eritrea OR Eswatini OR ‘’Equatorial Guinea" OR Ethiopia OR Gabon OR Ghana OR Guinea OR "Guinea-Bissau" OR Kenya OR Lesotho OR Liberia OR Madagascar OR Malawi OR Mali OR Mauritius OR Mauritania OR Mozambique OR Namibia OR Niger OR Nigeria OR ‘’Republic of the Congo’’ OR Rwanda OR ‘’Sao Tome and Principe’’ OR Senegal OR Seychelles OR "Sierra Leone" OR Somalia OR "South Africa" OR "South Sudan" OR Sudan OR Tanzania OR Togo OR Uganda OR Zambia OR Zimbabwe. ) AND ( “Screening Programme*” OR “Screening services” OR “Diabetic Eye examinations” OR “Diabetic retinal examinations” OR Screening OR “systematic screening” OR “mass screening” OR “opportunistic screening” OR “vision screening” OR “eye test” OR “Vision test” OR “Eye Assessment” OR “Vision Assessment” OR “retinal imaging” OR “slit lamp biomicroscopy” OR ophthalmoscopy OR “fundus photography” OR “Ocular coherence Tomography” ) AND ( “Diabetic eye disease” OR “Diabetic Retinopathy” OR “Diabetic macula oedema” OR “Diabetic macula edema” OR “Diabetic maculopathy” ) \| Expanders - Apply equivalent subjects Search modes - Boolean/Phrase \| Interface - EBSCOhost Research Databases Search Screen - Advanced Search Database - APA PsycInfo;CINAHL Complete \| 25 \| [Edit](javascript:__doPostBack('ctl00$ctl00$MainContentArea$MainContentArea$editControl$printHistory$HistoryRepeater$ctl01$linkEditSearch',''))S3 \| \| S2 \| ( ‘’sub Saharan Africa’’ OR ‘’sub-Saharan Africa” OR Africa OR Angola OR Benin or Botswana ‘’Burkina Faso’’ OR Burundi OR Cameroon OR ‘’Central Africa Republic’’ OR "Cape Verde" OR Chad OR Comoros OR ‘’Cote d’Ivoire’’ OR Djibouti OR ‘’Democratic Republic of the Congo’’ OR Eritrea OR Eswatini OR ‘’Equatorial Guinea" OR Ethiopia OR Gabon OR Ghana OR Guinea OR "Guinea-Bissau" OR Kenya OR Lesotho OR Liberia OR Madagascar OR Malawi OR Mali OR Mauritius OR Mauritania OR Mozambique OR Namibia OR Niger OR Nigeria OR ‘’Republic of the Congo’’ OR Rwanda OR ‘’Sao Tome and Principe’’ OR Senegal OR Seychelles OR "Sierra Leone" OR Somalia OR "South Africa" OR "South Sudan" OR Sudan OR Tanzania OR Togo OR Uganda OR Zambia OR Zimbabwe. ) AND ( “Screening Programme*” OR “Screening services” OR “Diabetic Eye examinations” OR “Diabetic retinal examinations” OR Screening OR “systematic screening” OR “mass screening” OR “opportunistic screening” OR “vision screening” OR “eye test” OR “Vision test” OR “Eye Assessment” OR “Vision Assessment” OR “retinal imaging” OR “slit lamp biomicroscopy” OR ophthalmoscopy OR “fundus photography” OR “Ocular coherence Tomography” ) \| Expanders - Apply equivalent subjects Search modes - Boolean/Phrase \| Interface - EBSCOhost Research Databases Search Screen - Advanced Search Database - APA PsycInfo;CINAHL Complete \| 6,817 \| [Edit](javascript:__doPostBack('ctl00$ctl00$MainContentArea$MainContentArea$editControl$printHistory$HistoryRepeater$ctl02$linkEditSearch',''))S2 \| \| S1 \| ‘’sub Saharan Africa’’ OR ‘’sub-Saharan Africa” OR Africa OR Angola OR Benin or Botswana ‘’Burkina Faso’’ OR Burundi OR Cameroon OR ‘’Central Africa Republic’’ OR "Cape Verde" OR Chad OR Comoros OR ‘’Cote d’Ivoire’’ OR Djibouti OR ‘’Democratic Republic of the Congo’’ OR Eritrea OR Eswatini OR ‘’Equatorial Guinea" OR Ethiopia OR Gabon OR Ghana OR Guinea OR "Guinea-Bissau" OR Kenya OR Lesotho OR Liberia OR Madagascar OR Malawi OR Mali OR Mauritius OR Mauritania OR Mozambique OR Namibia OR Niger OR Nigeria OR ‘’Republic of the Congo’’ OR Rwanda OR ‘’Sao Tome and Principe’’ OR Senegal OR Seychelles OR "Sierra Leone" OR Somalia OR "South Africa" OR "South Sudan" OR Sudan OR Tanzania OR Togo OR Uganda OR Zambia OR Zimbabwe. \| Expanders - Apply equivalent subjects Search modes - Boolean/Phrase \| Interface - EBSCOhost Research Databases Search Screen - Advanced Search Database - APA PsycInfo;CINAHL Complete \| 168,645 \| [Edit](javascript:__doPostBack('ctl00$ctl00$MainContentArea$MainContentArea$editControl$printHistory$HistoryRepeater$ctl03$linkEditSearch',''))S1 \| |
| --- | --- | --- | --- | --- | --- | --- | --- | --- | --- | --- | --- | --- | --- | --- | --- | --- | --- | --- | --- | --- | --- | --- | --- | --- | --- | --- | --- | --- | --- | --- |
|     |

[Top of Page](javascript:window.scrollTo(0,0);)

- [Download on the App Store](https://apps.apple.com/us/app/ebsco-mobile/id1473281170)
- [Get it on Google Play](https://play.google.com/store/apps/details?id=com.ebsco.ebscomobile)
- [EBSCO Connect](https://connect.ebsco.com/)
- [Privacy Policy](https://www.ebsco.com/company/privacy-policy)
- [A/B Testing](https://www.ebsco.com/conversion-testing-statement)
- [Terms of Use](https://www.ebsco.com/terms-of-use)
- [Copyright](https://www.ebsco.com/terms-of-use)
- [Cookie Policy](https://www.ebsco.com/cookie-policy)
- [Manage my Cookies](https://web.p.ebscohost.com/Legacy/Views/UserControls/Ehost/)

© 2023 EBSCO Industries, Inc. All rights reserved.

Bottom of Form
