# Supplementary material for: Factors associated with the uptake and utilisation of diabetic retinopathy screening services in sub-Saharan Africa: A scoping review
Source: PLoS One. 2024 Dec 13;19(12):e0315367. doi: 10.1371/journal.pone.0315367 (PMC11643260; doi:10.1371/journal.pone.0315367)
Supplement: S1 Appendix — (DOCX) [file pone.0315367.s002.docx]

**SCOPING REVIEW OF THE FACTORS ASSOCIATIED WITH THE UPTAKE AND UTILIZATION OF DIABETIC RETINOPATHY SCREENING SERVICES IN SUB-SAHARAN AFRICA**

**INTRODUCTION**

Diabetes Mellitus is a long-term metabolic condition marked by high blood glucose levels which could seriously harm the heart, blood vessels, eyes, nerves, and kidneys over time. It typically presents with symptoms of polyuria or frequent urination, polydipsia (increased thirst) and increased hunger also known as polyphagia. There are two broad categories namely Type 1 Diabetes (formerly known as Juvenile-onset or insulin dependent Diabetes) and the more prevalent Type 2 Diabetes or Adult-Onset Diabetes.(Deshmukh *et al.* 2015). Diabetic retinopathy, on the other hand, is a sight threatening disease condition which affects tiny blood vessels of the retina in people with long standing diabetes mellitus ultimately leading to vision loss. It can be loosely classified into non-proliferative and proliferative stages with distinct clinical features.(Nijalingappa and Sandeep 2015). In 2003, the international classification of Diabetic Retinopathy (ICDR) introduced the International Clinical Disease Severity Scale for Diabetic retinopathy providing a detailed 5-stage classification of the condition as follows: no apparent retinopathy stage; mild non-proliferative diabetic retinopathy (NPDR); moderate NPDR; severe NPDR; and Proliferative Diabetic Retinopathy (PDR) and 3 categories of Diabetic macular edema: mild DME, moderate DME, Severe DME. (Wu *et al.* 2013). Other forms of classification that have been used in the past include the modified Airlie House classification used in Diabetic Retinopathy Study (DRS) and the Early Treatment Diabetic Retinopathy Study (ETDRS). (Das *et al.* 2021)

Diabetic retinopathy remains a prominent consequence of Diabetes mellitus and a major contributor to the growing burden of avoidable blindness in adult working populations globally. In 2021, the International Diabetes Federation (IDF) estimated that 537 million adults worldwide between the ages of 20 to 79 had diabetes and this figure is projected to increase significantly by 2045 (IDF 2021). Whilst laudable advancements have been made with respect to screening, diagnosis and management of Diabetes and its attendant complications in high income countries, there has been very little progress made in countries with low income resulting in a higher prevalence of Diabetic Retinopathy in these settings.(Teo *et al.* 2021). The propensity to develop retinopathy is intrinsically linked to the duration of Diabetes. Unfortunately, in low-resources settings like Africa, Diabetes is often diagnosed after it has markedly progressed, with the result that about 25% of people with type 2 diabetics and 9.5% of those with type 1 diabetics may already have developed Diabetic retinopathy at the time of diagnosis.(Levy and Jotkowitz 2010). Based on the foregoing, evaluating the present state of Diabetic retinopathy screening services in sub-Saharan Africa against the backdrop of the five A’s model of accessibility (Approachability; Acceptability; Availability and accommodation; Affordability; Appropriateness) is paramount to ensure efficient, and cost-effective screening services are put in place and optimally utilized to facilitate early detection and timely intervention.(Levesque *et al.* 2013).

**REVIEW QUESTION**

What are the factors impacting the provision and uptake of Diabetic Retinopathy screening services for people living with Diabetes in sub-Saharan Africa?

**FRAMEWORK**: The Population, Concept and Context (PCC) framework for conducting scoping reviews recommended by Joanna Briggs Institute (JBI) in 2013 was used to develop our review question.(Khalil *et al.* 2021). Based on this method of evidence synthesis, we can deduce the following:

**Population of Interest:** People living with Diabetes.

**Concept**: Uptake and utilization of Diabetic retinopathy screening services.

**Context:** sub-Saharan Africa

**STUDY AIM**

This study aims to look at the contextual issues affecting the provision and utilization of screening services for Diabetic retinopathy in resource-poor settings in sub-Saharan Africa.

**REVIEW OBJECTIVES**

- To comprehensively map the extent of the existing body of literature on Diabetic retinopathy screening services in sub-Saharan Africa.
- To highlight the factors that impact the uptake and utilization of screening services from the consumer and provider standpoints.

**METHODS**

A scoping review will be conducted to answer the research question and shed light on the volume and scope of the studies that have been carried out and highlight any research gaps for future studies with respect to screening programmes for Diabetic Retinopathy in sub-Saharan Africa.

**METHODOLOGY**: This review will be conducted using the 5 steps outlined in the methodological framework by (Arksey and O'Malley 2005) which includes identifying the research question, identifying relevant studies, study selection, charting the data, collating, summarising, and reporting results. Additionally, in keeping with the updated framework by (Levac *et al.* 2010), we shall be adopting an iterative team approach during study selection and data abstraction phases of the review as well as ensuring our findings are adequately conveyed to policymakers to inform evidence-based healthcare practices.

**SEARCH STRATEGY**: To identify relevant studies for the review, we will be searching electronic databases by signing into the EBSCO host platform on the University of Limerick Glucksman Library website. Electronic research databases to be searched include MEDLINE/PubMed from the National Library of Medicine, Embase, PsychINFO, Web of Science, CINHAL Complete, Africa Journals Online.

No publication time limits will be applied to the search. Each database will be searched separately to ensure a thorough and systematic procedure is followed.

*Keywords for the search include:*

***“Diabetes eye disease” OR “Diabetic Retinopathy” OR “Diabetic macula oedema” OR “Diabetic macula edema” OR “Diabetic maculopathy”***

*AND*

***“Screening Programme*” OR “Screening services” OR “Diabetic Eye examinations” OR “Diabetic retinal examinations” OR Screening OR “systematic screening” OR “mass screening” OR “opportunistic screening” OR “vision screening” OR “eye test” OR “Vision test” OR “Eye Assessment” OR “Vision Assessment” OR “retinal imaging” OR “slit lamp biomicroscopy” OR ophthalmoscopy OR “fundus photography” OR “Ocular coherence Tomography.***”

*AND*

***‘’Sub Saharan Africa’’ OR ‘’sub-Saharan Africa, OR Angola OR Benin OR Botswana OR ‘’Burkina Faso’’ OR Burundi OR Cameroon OR ‘’Central Africa Republic’’ OR “Cape Verde” OR Chad OR Comoros OR ‘’Cote d’Ivoire’’ OR Djibouti OR ‘’Democratic Republic of the Congo’’ OR Eritrea OR Eswatini OR ‘’Equatorial Guinea” OR*** ***Ethiopia OR Gabon OR Ghana OR Guinea OR “Guinea-Bissau” OR Kenya OR Lesotho OR Liberia OR Madagascar OR Malawi, OR Mali OR Mauritius OR Mauritania OR Mozambique OR Namibia OR Niger OR Nigeria OR ‘’Republic of the Congo’’ OR Rwanda OR ‘’Sao Tome and Principe’’ OR Senegal OR Seychelles OR “Sierra Leone” OR Somalia OR “South Africa” OR “South Sudan” OR Sudan OR Tanzania OR Togo OR Uganda OR Zambia OR Zimbabwe.***

Searches will be run for these terms in the title, abstract and full-text fields for a more comprehensive search. To guide our search properly and increase the chances of retrieving high quality journal articles, we will search for related terms for our key concept “Diabetic retinopathy screening services” under the medical subject headings for each database MeSH terms for MEDLINE, CINAHL subheadings for CINAHL complete and so on, using a combination of Boolean operators (AND, OR, NOT) and other search terms to narrow or broaden the search as required. We shall also be looking at keywords in the studies we find and hand searching their references lists and citations to expand, refine and modify our search terms. To widen our search range, we also plan on contacting field experts in the health sector and academia. Considering, the amount of iteration involved during this stage of the review, utmost care will be taken to save our search strings after each session to enable us keep track of all our queries. Furthermore, we will be using Endnote as our reference manager to aid in the gathering of references and tracking of relevant publications.

**STUDY SELECTION:** Articles found on the electronic research databases and those identified through other sources will be scrutinized for duplicates and these will be removed manually. Studies will then be screened for relevance, and methodological rigor after reviewing titles and abstracts using the software application Rayyan. Subsequently, full-text articles will be assessed and selected if the eligibility criteria (outlined below) are satisfied. Studies will be displayed using the Preferred Reporting Items for Systematic review and Meta-Analysis (PRISMA) flow diagram in figure 1. This is necessary to promote transparency, accountability, and reproducibility in the selection process. Reasons for exclusion of articles at the full-text stage will equally be appropriately provided and documented.


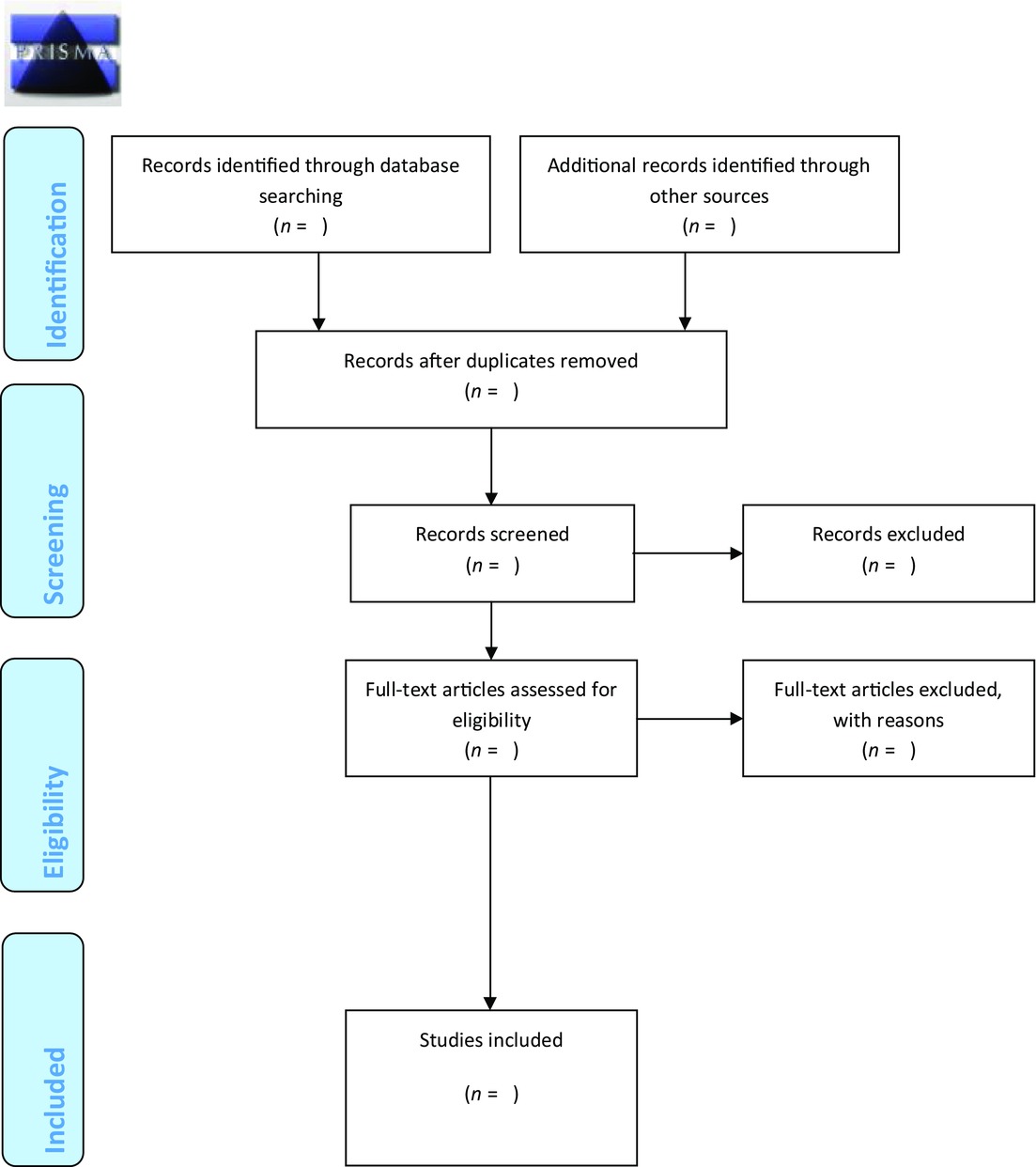


**Figure 1: PRISMA FLOW DIAGRAM**

**INCLUSION CRITERIA:**

- Studies that assessed the factors associated with Diabetic retinopathy screening from consumers and/or service providers perspectives.
- Studies using quantitative, qualitative, or mixed methods to assess Diabetic retinopathy screening programmes or services.
- Articles that included participants and/or service providers in an existing diabetes/DR screening program.
- Population or hospital-based studies conducted in sub-Saharan Africa.
- Articles published in English**.**

**EXCLUSION CRITERIA**

- Studies that do not involve human subjects living with Diabetes.
- Studies evaluating hurdles to eye care in general, without focusing on Diabetic retinopathy screening.
- Editorials, Case reports, opinion papers, conference abstracts and review protocols.
- Studies focusing solely on prevalence, diagnosis, medical, or surgical/para-surgical management of Diabetic retinopathy.
- Research assessing screening obstacles for Diabetes Mellitus, without mentioning screening hurdles for Diabetic retinopathy specifically.
- Articles not published in English.
- Studies not conducted in sub–Saharan Africa

**DATA CHARTING**

The full text of the included studies for the scoping review will be read by the reviewers and relevant data will be extracted.

Information to be extracted include:

author, title, year of publication, name and type of journal, study location, aims and objectives of the research, sample size such as number of people living with diabetes in the study sample, number of people diagnosed with diabetic retinopathy and their stages of progression, number of service providers evaluated, number of healthcare facilities assessed, study design and research methods used, key research findings, interventions, conclusions, and recommendations. Microsoft Excel will be used for charting the data and the entire process will be conducted and reviewed independently by the researchers. This will also be piloted on a few of the included studies and subsequently refined as needed.

**COLLATING, SUMMARIZING, AND REPORTING FINDINGS**

In line with the (Arksey and O’Malley, 2005) methodological framework, a narrative account of key findings from the existing literature will be summarized after collating, and analysing the data extracted for emerging themes. This thematic analysis will also be presented descriptively in a tabular format so that all relevant stakeholders and researchers interested in the topic can easily access key study findings. Studies will be reported according to the Preferred Reporting Items of Systematic reviews and Meta-analysis PRISMA-ScR extension guidelines of 2018 with the subsequent creation of a manuscript.

**LIMITATIONS**

Given the sheer volume of literature included, a scoping review by default will not allow for a formal critical appraisal of the studies with tools such as the Newcastle-Ottawa quality assessment scale. Since our focus is to determine the extent and nature of the existing body of evidence within a specified timeframe, there may be issues related to time and resources constraints. Other possible sources of bias might come from small sample sizes used in the included studies and socio-demographic factors peculiar to certain subsets of the population which could make it difficult to generalise the findings to the entire population. There might also be language bias since, we will be including studies published in English only in the review.

**REFERENCES**

Arksey, H. and O'Malley, L. (2005) 'Scoping studies: towards a methodological framework', *International journal of social research methodology*, 8(1), 19-32.

Das, T., Takkar, B., Sivaprasad, S., Thanksphon, T., Taylor, H., Wiedemann, P., Nemeth, J., Nayar, P.D., Rani, P.K. and Khandekar, R. (2021) 'Recently updated global diabetic retinopathy screening guidelines: commonalities, differences, and future possibilities', *Eye*, 35(10), 2685-2698, available: <http://dx.doi.org/10.1038/s41433-021-01572-4>.

Deshmukh, C.D., Jain, A. and Nahata, B. (2015) 'Diabetes mellitus: a review', *Int. J. Pure Appl. Biosci*, 3(3), 224-230.

IDF, I.D.F. (2021) *About Diabetes: Diabetes facts and figures*, available: <https://www.idf.org/aboutdiabetes/what-is-diabetes/facts-figures.html> [accessed 5th, December, 2022].

Khalil, H., Peters, M.D., Tricco, A.C., Pollock, D., Alexander, L., McInerney, P., Godfrey, C.M. and Munn, Z. (2021) 'Conducting high quality scoping reviews-challenges and solutions', *Journal of clinical epidemiology*, 130, 156-160.

Levac, D., Colquhoun, H. and O'Brien, K.K. (2010) 'Scoping studies: advancing the methodology', *Implementation science*, 5(1), 1-9.

Levesque, J.F., Harris, M.F. and Russell, G. (2013) 'Patient-centred access to health care: conceptualising access at the interface of health systems and populations', *Int J Equity Health*, 12, 18, available: <http://dx.doi.org/10.1186/1475-9276-12-18>.

Levy, J. and Jotkowitz, A.B. (2010) 'Diabetes in Africa: Screening for diabetic retinopathy', *European Journal of Internal Medicine*, 21(3), 145-146, available: <http://dx.doi.org/https://doi.org/10.1016/j.ejim.2010.02.010>.

Nijalingappa, P. and Sandeep, B. (2015) 'Machine learning approach for the identification of diabetes retinopathy and its stages', in *2015 International Conference on Applied and Theoretical Computing and Communication Technology (iCATccT)*, 29-31 Oct. 2015, 653-658, available: <http://dx.doi.org/10.1109/ICATCCT.2015.7456965>.

Teo, Z.L., Tham, Y.-C., Yu, M., Chee, M.L., Rim, T.H., Cheung, N., Bikbov, M.M., Wang, Y.X., Tang, Y., Lu, Y., Wong, I.Y., Ting, D.S.W., Tan, G.S.W., Jonas, J.B., Sabanayagam, C., Wong, T.Y. and Cheng, C.-Y. (2021) 'Global Prevalence of Diabetic Retinopathy and Projection of Burden through 2045: Systematic Review and Meta-analysis', *Ophthalmology*, 128(11), 1580-1591, available: <http://dx.doi.org/https://doi.org/10.1016/j.ophtha.2021.04.027>.

Wu, L., Fernandez-Loaiza, P., Sauma, J., Hernandez-Bogantes, E. and Masis, M. (2013) 'Classification of diabetic retinopathy and diabetic macular edema', *World J Diabetes*, 4(6), 290-4, available: <http://dx.doi.org/10.4239/wjd.v4.i6.290>.
